# Graph visualization efficiency of popular web-based libraries

**DOI:** 10.1186/s42492-025-00193-y

**Published:** 2025-05-08

**Authors:** Xin Zhao, Xuan Wang, Xianzhe Zou, Huiming Liang, Genghuai Bai, Ning Zhang, Xin Huang, Fangfang Zhou, Ying Zhao

**Affiliations:** 1 School of Computer Science and Engineering, Central South University, Changsha, 410083 Hunan China; 2 QAX Security Center, Qi An Xin Technology Group Inc., Beijing, 100044 China

**Keywords:** Graph visualization, Node-link diagram, Web-based visualization, Visualization library

## Abstract

Web-based libraries, such as D3.js, ECharts.js, and G6.js, are widely used to generate node-link graph visualizations. These libraries allow users to call application programming interfaces (APIs) without identifying the details of the encapsulated techniques such as graph layout algorithms and graph rendering methods. Efficiency requirements, such as visualizing a graph with 3k nodes and 4k edges within 1 min at a frame rate of 30 fps, are crucial for selecting a proper library because libraries generally present different characteristics owing to the diversity of encapsulated techniques. However, existing studies have mainly focused on verifying the advantages of a new layout algorithm or rendering method from a theoretical viewpoint independent of specific web-based libraries. Their conclusions are difficult for end users to understand and utilize. Therefore, a trial-and-error selection process is required. This study addresses this gap by conducting an empirical experiment to evaluate the performance of web-based libraries. The experiment involves popular libraries and hundreds of graph datasets covering node scales from 100 to 200k and edge-to-node ratios from 1 to 10 (including complete graphs). The experimental results are the time costs and frame rates recorded using the libraries to visualize the datasets. The authors analyze the performance characteristics of each library in depth based on the results and organize the results and findings into application-oriented guidelines. Additionally, they present three usage cases to illustrate how the guidelines can be applied in practice. These guidelines offer user-friendly and reliable recommendations, aiding users in quickly selecting the desired web-based libraries based on their specific efficiency requirements for node-link graph visualizations.

**Supplementary Information:**

The online version contains supplementary material available at 10.1186/s42492-025-00193-y.

## Introduction

Node-link graph visualization is widely used to provide a profound understanding of the global and local structures of a graph [1–7]. Implementing a node-link graph visualization involves several advanced techniques, including graph layout and graph rendering [8, 9]. Web-based graph visualization libraries [10–12] such as D3.js, ECharts.js, and G6.js have encapsulated graph layout algorithms and graph rendering methods, allowing users to easily generate node-link visualizations by selecting application programming interfaces (APIs) without identifying the details of the advanced techniques. Therefore, using these libraries is the preferred option for end users who regularly engage in graph visualization tasks, providing a convenient and efficient way to generate node-link visualizations.

Web-based graph visualization libraries present diverse characteristics [13]. Through preliminary surveys with end users, including front-end engineers, data analysts, students, and academics, we investigated the factors they consider when selecting a visualization library. Based on their feedback, we identified three main factors that were most frequently mentioned and prioritized by the end users: functional requirements, programming complexity, and efficiency requirements. Functional requirements can be easily confirmed by viewing library documentation and online examples. Low-level encapsulated libraries, such as D3.js, tend to have higher programming complexity, whereas high-level libraries, such as ECharts.js and G6.js, have lower programming complexity. However, the confirmation of efficiency requirements requires considerable trial and error owing to three complex, interrelated factors: (1) time cost and frame rate, (2) graph layout algorithm and graph rendering method, and (3) numbers of nodes and edges.

Specifically, time cost refers to the duration required for a library to generate a node-link graph visualization result [8, 11]. Frame rate is the number of visualization images displayed per second in a browser [14]. The time cost and frame rate are mainly determined by the graph layout algorithm and rendering method [8, 14]. Most graph layout algorithms require hundreds of iterative calculations to determine node positions [15–19]. A rendering process follows each iteration. Each cycle of the layout iteration and rendering process produces one visualization image. Therefore, graph layout iterations and rendering processes contribute to the time cost and frame rate. A low time cost enables users to quickly obtain a stable graph visualization. A high frame rate ensures a smooth graph-visualization animation during the iteration calculations [20].

Typically, only information on the number of nodes and edges is available to users, without a deep understanding of graph layout algorithms and rendering methods, when selecting a library to meet their efficiency requirements. However, visualizing the same graph data using different libraries generally results in varied time costs and frame rates [14, 21] because these libraries adopt different layout algorithms and support various rendering methods. Moreover, the relationship between graph size and time cost/frame rate is intricate. Therefore, users must conduct experiments to verify the library that satisfies their efficiency requirements. However, the trial-and-error process is time-consuming and tedious.

To the best of the authors knowledge, systematic and application-oriented guidance for end users in choosing a web-based graph visualization library from the perspective of efficiency requirements is lacking owing to three main reasons. First, library designers tend to provide the functional features and usage examples of a library; however, they overlook specifying its efficiency advantages in detail [22]. Second, software engineers may share their experience of using one or two libraries on a few specific graphs; however, they have insufficient time to systematically evaluate library efficiency. Finally, although researchers have investigated the performance of existing graph layout algorithms and rendering methods [23, 24], they have focused on demonstrating the advantages of the newly proposed layout algorithm or rendering method. Their efforts are from a source-code level and a theoretical viewpoint, independent of specific popular web-based libraries, making it difficult for end users to understand and utilize their findings.

To address this gap, this study evaluated the performance of web-based graph visualization libraries through a systematic, controlled experimet. The experiment involved three popular libraries: D3.js, ECharts.js, and G6.js, and three rendering methods: scalable vector graphics (SVG), Canvas, and web graphics library (WebGL), resulting in seven experimental library entries: D3-SVG, D3-Canvas, D3-WebGL, ECharts-SVG, ECharts-Canvas, G6-SVG, and G6-Canvas. The study selected 481 graph datasets with node scales ranging from 100 to 200k and edge-to-node ratios ranging from 1 to 10 (including complete graphs (CGs)). Among them, 156 datasets were sourced from real-world scenarios and 325 datasets were synthetic to overcover the node scales and edge-to-node ratios. The experiment was conducted on a mainstream desktop computer equipped with a common browser and operating system. The time cost and frame rate for generating node-link diagrams using the libraries and datasets were recorded during the experiment.

The time cost and frame rate results were thoroughly analyzed. Performance trends and differences in the experimental libraries with increasing node scales and edge-to-node ratios were identified. The factors contributing to these findings were summarized. The experimental results and findings were reorganized into application-oriented guidelines, and three usage cases are provided to illustrate how the guidelines can be applied for practical use. A end user with specific efficiency requirements (e.g., graph scales, time cost limits, and acceptable frame rates) for generating node-link diagrams can identify suitable libraries by consulting our guidelines. For example, if a user needs to visualize a graph with 3k nodes at an edge-to-node ratio of 1 within 1 min while maintaining a frame rate above 30 frames per second (fps), they can see that D3-WebGL and D3-Canvas meet these requirements.

In summary, this study presents a systematic evaluation of the performance of popular web-based graph visualization libraries, an in-depth analysis of the performance trends and differences in libraries, and application-oriented guidelines to help end users identify suitable libraries that meet their specific efficiency requirements for quickly generating node-link graph visualizations.

### Graph visualization libraries

Graph visualization libraries can be categorized as either desk-or web-based. Desk-based libraries are integrated into desktop applications for local data visualization. Popular examples of desk-based libraries include Tableau, Matplotlib, JavaFX, and GTK+ [22]. These libraries are commonly used for visualization scenarios that require local deployment and offline data presentation. Conversely, web-based libraries allow users to generate graph visualizations in JavaScript-supported web browsers. Popular web-based libraries include D3.js, G6.js, ECharts.js, Sigma.js, Chart.js, NetV.js and, Linkurious.js [10–12, 14, 25–27]. Web-based libraries offer greater accessibility, cross-platform compatibility, and easier deployment than desktop-based libraries, making them the preferred choice for most users. This study focuses on web-based libraries.

### Graph layout algorithms

Graph layout algorithms include force-directed [15–19], constraint-based [28–30], and dimensionality reduction [31–34] algorithms. Force-directed algorithms simulate node interaction forces using physical models such as springs and particles to generate node-link visualizations. Representative algorithms include Spring [15], Fruchterman-Reingold [16], Spring Embedder [17], and ForceAtlas2 [18]. Although force-directed algorithms produce visually appealing and easily understandable layout results with low implementation difficulty, they have high computational complexity and difficulty in achieving globally optimal layouts. Most graph visualization libraries, including those selected in this study utilize these algorithms [10–12, 14, 25–27]. Constraint-based layout algorithms position nodes and edges based on specified constraints such as node distances and positional limitations. Representative constraint-based layout algorithms include grid, circular, and hierarchical layouts [28–30]. These algorithms offer good flexibility because they can achieve customized layout results through user-defined constraints. However, the design of suitable and conflict-free constraints can be challenging. Dimensionality reduction algorithms project nodes into a two-dimensional space based on their characteristics in a high-dimensional space. Classic dimensionality reduction algorithms include high-dimensional embedding [31], pivot multidimensional scaling [32], and DRGraph [33]. These algorithms are known for their high layout speeds. However, the projection process may lead to an improper representation of local graph structures and a low-readability layout result.

### Graph rendering methods

Web-based graph visualization libraries rely on native browser graphics methods (i.e., rendering methods) to convert data into visible graphical representations. SVG, Canvas, and WebGL are the three main methods used to natively render graphics [24, 35, 36]. Each rendering method has its own advantages. The SVG method uses geometric shapes, paths, and texts to render graphs, which can be scaled and resized without losing quality and is ideal for creating interactive visualizations that require precise control over individual elements. Canvas directly manipulates pixel-based data on a browser surface, making it suitable for rendering animations and other scenes with low interaction requirements. WebGL is based on the Open Graphics Library [37] and enables users to create graphics processing units (GPUs) to accelerate visualizations directly in the browser. It is commonly used to create immersive three-dimensional games, virtual reality experiences, and scientific simulations [38–43]. Most web-based visualization libraries are built using at least one of the three rendering methods. For example, G6.js and ECharts.js stably support the SVG and Canvas methods, whereas D3.js stably supports all three rendering methods.

## Methods

This study systematically evaluates the efficiency of popular web-based graph visualization libraries in terms of time cost and frame rate. The findings could guide end users in quickly selecting a library that meets their graph visualization efficiency requirements based on the size of the graph dataset. Therefore, the authors conducted a controlled experiment using popular web-based graph visualization libraries and a considerable number of various graph datasets. The details of the experimental design are presented below.

### Graph visualization library selection

D3.js, G6.js, and ECharts.js libraries were used in the experiment based on three considerations. First, the three libraries are popular open-sourced web-based libraries that support node-link visualizations. They obtained GitHub stars of 108k, 58.7k, and 10.7k, respectively, as of April 2024. Second, each supported at least two rendering methods, facilitating an efficiency comparison between the rendering methods. Finally, they were created for diverse target audiences to ensure that the experimental results can benefit a wide range of users. D3.js is tailored for professional developers with high customization requirements because it provides numerous APIs for manipulating the underlying data items and graphical elements. However, D3.js has a relatively high development difficulty and a steep learning curve. G6.js integrates many advanced graph visualization functions and algorithms, making it suitable for senior data analysts with specialized graph analysis requirements. Its development and learning difficulties are lower than those in D3.js. ECharts.js targets junior data analysts and software developers with limited visualization knowledge and experience, with its simple and user-friendly APIs making it the easiest to use among the three libraries. The following versions were used in this study: version 5.16.0 for D3.js, released in April 2020; version 5.4.2 for ECharts.js, released in May 2023; and version 4.8.7 for G6.js, released in February 2023.

Because the rendering method considerably affects graph visualization efficiency, it is stipulated that the experimental unit is a pair of a web-based graph visualization library and one of its supported rendering methods. ECharts.js and G6.js supported the SVG and Canvas methods, whereas D3.js supported the SVG, Canvas, and WebGL methods. Consequently, seven experimental library entries were obtained: D3-SVG, D3-Canvas, D3-WebGL, ECharts-SVG, ECharts-Canvas, G6-SVG, and G6-Canvas. Notably, the use of native WebGL codes to implement D3-WebGL is difficult. Developers generally adopted additional libraries (i.e., NetV.js or Three.js) to achieve the WebGL rendering part in D3.js. The authors selected NetV.js (version v1.1.11 released in May 2023) [14] for the experiment because it was designed for large-scale graph visualization.

### Experimental library parameter configuration

Libraries are used to generate the node-link visualizations required to configure the node/edge style, canvas, and layout algorithm parameters.

For the node/edge style parameters, the authors set the node diameter and edge width of each library to 1 pixel. The nodes and edges have no borders or text labels. For the canvas parameter, they set the width and height of the canvas to 3k $\times$ 3k pixels.

For the layout algorithms, each library used its default force-directed layout algorithm and parameter values. The main parameter values are as follows: strength, theta, alphaDecay, and alphaMin with values of -30, 0.9, 0.0228, and 0.001 for the D3-SVG, D3-Canvas, and D3-WebGL libraries, respectively. For the G6-SVG and G6-Canvas libraries, the main parameters included node/edge strength, alpha, alphaDecay, and alphaMin, with parameter values of null, 0.3, 0.028, and 0.001, respectively. For the ECharts-SVG and ECharts-Canvas libraries, the main parameters included repulsion, gravity, and friction, with parameter values of 50, 0.1, and 0.6, respectively. For the layout algorithm iteration count, the authors set the parameter value for each library to 200 times. This parameter determines the number of iterations a layout algorithm performs before stopping the computation. Empirically, 200 iterations yield relatively stable layout results. To reduce the length of the paper, detailed parameters are provided in the Supplementary Material.

### Graph dataset

The dataset selection for this experiment was based on three considerations: (1) it should cover a wide range of node scales, (2) cover a various edge scales, and (3) involve diverse graph types.

The node scales of the experimental graph datasets ranged from 100 to 200k, covering most application scenarios of node-link diagrams. Pilot experiments indicated that WebWorker multithreading or GPU-based parallel computing was required to generate the node-link visualization of a graph with more than 200k nodes on a mainstream desktop computer within a short time. The authors divided the range of node scales into 47 levels with four progressive intervals. Specifically, 10 node-scale levels were set from 100 to 1k nodes with a fixed interval of 100 nodes; 9 node-scale levels were set from 1k to 10k nodes with an interval of 1k nodes; 18 node-scale levels were set from 10k to 100k nodes with an interval of 5k nodes; and 10 node-scale levels were set from 100k to 200k nodes with an interval of 10k nodes. This division method can obtain fine-grained experimental results for small- and medium-sized graphs commonly encountered by most users, whereas coarse-grained experimental results for uncommon large-sized graphs reduce the computational burden.

The edge scale of the experimental graph dataset was measured using the edge-to-node ratio (i.e., the number of edges divided by the number of nodes). Ten edge-scale levels were set from 1 to 10 edge-to-node ratios at fixed intervals for two reasons. First, users typically regard the number of nodes, instead of the number of edges, as the main consideration for the size of a graph dataset. Therefore, the authors stipulated that the number of nodes and edge-to-node ratio would be the primary and secondary variables in determining the size of an experimental graph dataset, which is consistent with user habits and could also simplify the experimental design. Second, the edge-to-node ratio is an important metric for distinguishing different network types. For example, the edge-node ratios of sparse networks [44] (e.g., transportation, biological, and logistics networks) and small-world networks [45] (e.g., brain networks, power grids, and social networks) are approximately 1 and 3, respectively. The edge-node ratios of scale-free networks [46] (e.g., social networks, internet topology, and scientific collaboration networks) range from 1 to 5, with a few reaching 6 to 10 and very few exceeding 10 [47]. Therefore, using the edge-to-node ratio and 10 edge-scale levels can help users understand the efficiency advantages of graph visualization libraries from the perspective of network types.

Each pair of a node-scale level and an edge-to-node ratio had a corresponding graph dataset. In total, 470 datasets were established (47 node-scale levels $\times$ 10 edge-to-node ratios). Moreover, the authors used CGs and created a CG dataset for each of the 11 node-scale levels, ranging from 100 to 2k nodes. Pilot experiments demonstrated that CGs exceeding 2k nodes would have over ten million edges, making it extremely difficult to generate visualizations on a mainstream desktop computer in a short time. Consequently, 481 graph datasets were established. Among them, 156 were sourced from real-world networks, including social, citation, biological, and internet networks. Relevant public repositories include network repositories, UCI network data repositories, Pajek datasets, and the Stanford SNAP repository [48–51]. The authors fine-tuned 114 of the 156 real-world network datasets and manually synthesized 325 datasets to ensure that each node-scale level and edge-to-node ratio pair contained one dataset. Additionally, the node-link diagrams generated from each dataset were simple graphs with attribute-free, content-agnostic edges and nodes. They utilized the Python NetworkX library [52] to synthesize datasets, which offered flexible functions and parameters (including the target node scale and edge-to-node ratio) to generate desired graph datasets.

### Apparatus

A mainstream desktop computer was used to ensure the universality of the experimental results. The computer ran on the Windows 10 operating system, equipped with a $12^{th}$ GEN Intel(R) $Core^{TM}$ i7-12700 CPU @ 2.10GHz processor, 32.0GB of RAM, NVIDA GeForce RTX 3060, and a Samsung SSD 980 1TB hard drive. The authors used the Chrome browser version 106.0.5249.119 for the experiments.

### Procedure

The authors created a script to visualize each experimental dataset sequentially using each experimental library three times. They then recorded the time cost and frame rate for each visualization. The time cost is the time required to load a dataset into a library until 200 iterations of layout computing and rendering are completed. The authors recorded the overall time cost of graph visualization rather than the time cost for individual technical steps. This decision was based on the fact that end users often treat these libraries as a “black box” and are primarily concerned with how quickly the final visualization can be generated. For the D3-SVG, D3-Canvas, and D3-WebGL libraries, the time cost was measured by recording the time required for 200 tick events. For G6-SVG and G6-Canvas, the time difference between beforeRender and afterRender events was calculated. The time cost for the ECharts-SVG and ECharts-Canvas libraries was measured by recording the time required for 200 rendered events. Frame rate is the number of visualization images displayed per second in a browser. Each image was produced when a graph layout iteration and graph rendering cycle were completed. The frame rate was obtained using the browser’s requestAnimationFrame function. The maximum acceptable time to obtain graph visualization was 15 min. The time cost and frame rate results of a pair of datasets and libraries that failed to complete 200 iterations of layout computing and rendering within 15 min were discarded.

### Analysis approach

The raw experimental results were the raw time cost and raw frame rate recorded to obtain a visualization using each pair of graph dataset and graph visualization library. In total, 10,101 raw time costs and 10,101 raw frame rates (481 datasets $\times$ 7 libraries $\times$ 3 times) were collected. The authors conducted a set of statistical computations on the raw results to facilitate subsequent analyses. Tables 1 and 2 summarize some statistical computations results. Additional results are provided in the Supplementary Materials.

**Table 1.**
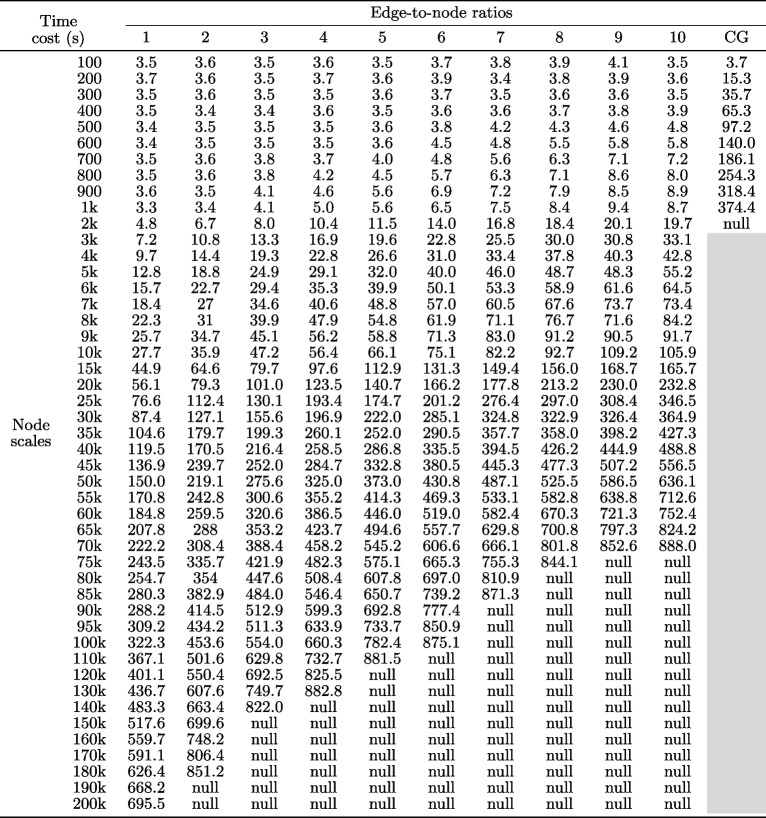
Time cost results of visualizing the 481 graph datasets (47 node-scale levels $\times$ 10 edge-to-node ratios + 11 node-scale levels $\times$ 1 special edge-to-node ratio of CG) using the D3-SVG library

A cell corresponds to the average time cost of visualizing the graph dataset three times. The row of the cell represents the node-scale of the dataset. The column of the cell represents the edge-to-node ratio of the dataset

For the raw time cost results, the average value of the three raw time costs recorded when visualizing a dataset using a library three times was computed. Table 1 summarizes the computation results for visualizing 481 datasets using the D3-SVG library. The cell in the second row and second column of Table 1 indicates that 3.6 s were spent when visualizing a dataset with a node scale of 200 (i.e., 200 nodes) and an edge-node ratio of 2 (i.e., 400 edges) using the D3-SVG library three times. Moreover, some cells had ‘null’ values, indicating that the corresponding dataset could not be visualized within 15 min using the D3-SVG library. Furthermore, CGs were only involved at node scales ranging from 100 to 2k nodes. Therefore, cells exceeding 2k nodes in the CG column were set with a gray background.

For the raw frame rate results, the authors computed the average value of the frame rates recorded when visualizing a dataset using a library three times. Table 2 displays the computation results for visualizing 481 datasets using the D3-SVG library. The cell in the second row and tenth column of Table 2 indicates that an average frame rate of 55.8 fps was obtained for visualizing a dataset with a node scale of 200 (i.e., 200 nodes) and an edge-to-node ratio of 10 (i.e., 2k edges) using the D3-SVG library three times. To reduce the paper length, the frame rate results of the other edge-to-node ratios are provided in the Supplementary Material.

**Table 2:**
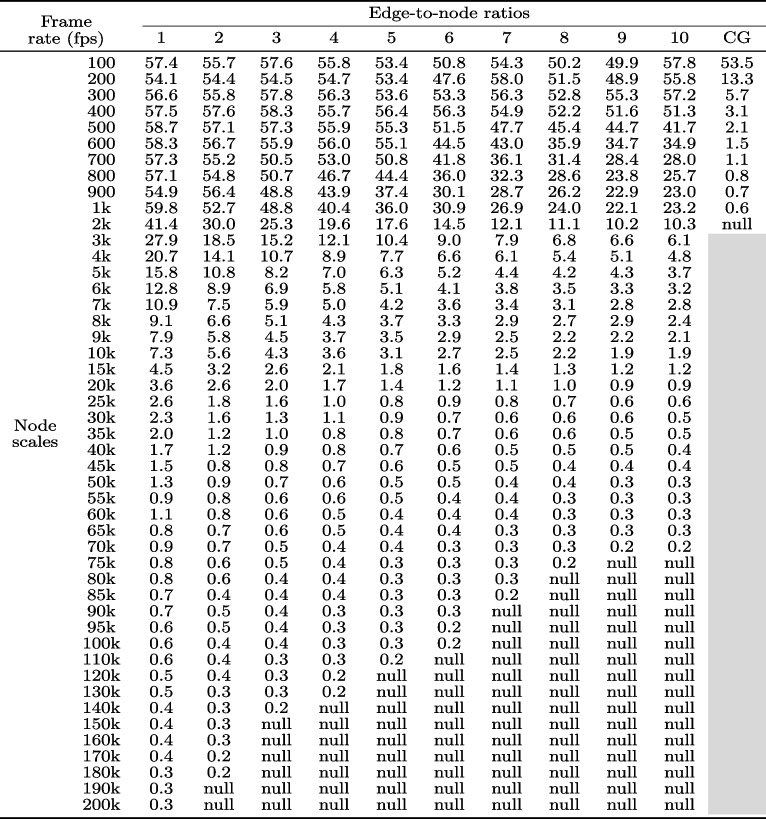
Frame rate results of visualizing the 481 graph datasets (47 node-scale levels $\times$ 10 edge-to-node ratios + 11 node-scale levels $\times$ 1 special edge-to-node ratio of CG) using the D3-SVG library

A cell corresponds to the average frame rate of visualizing a graph dataset three times. The row of the cell represents the node scale of the dataset. The column of the cell represents the edge-to-node ratio of the dataset

**Fig. 1:**
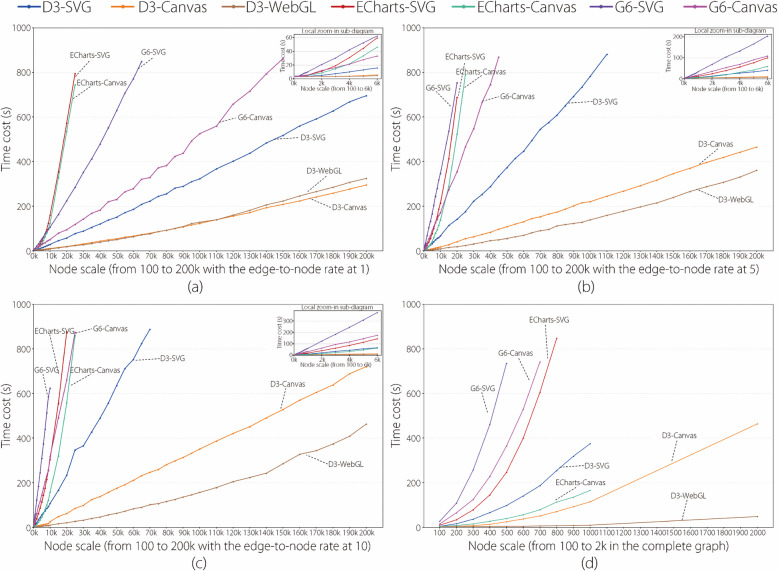
Line-chart visualizations of time cost results of all the libraries with all the node scales and edge-to-node ratios of 1 (**a**), 5 (**b**), 10 (**c**), and CG (**d**). In each line-chart diagram, the* x*-axis denotes the node scale, *y*-axis denotes the time cost, a colored line represents a library, a point on a line represents the time cost of visualizing a dataset using a library, and the top-right sub-diagram is the zoom-in view of the area of node scales from 100 to 6k

**Fig. 2:**
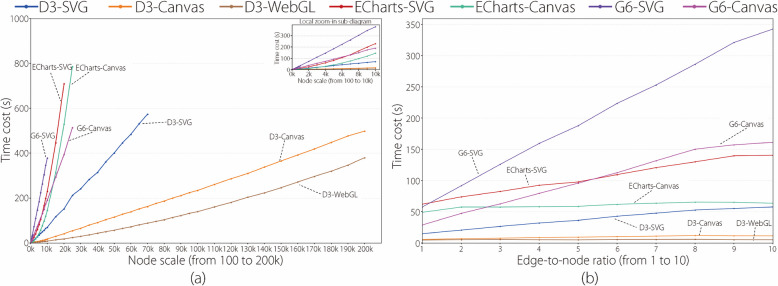
**a** Line-chart visualization of time cost results of all the libraries with the node scales from 100 to 200k. The *x*-axis denotes the node scale, *y*-axis denotes the time cost, a colored line represents a library, a point on a line represents the average time cost of visualizing the ten graph datasets (corresponding to the ten edge-to-node ratios from 1 to 10, respectively) at a node scale, and the top-right sub-diagram is the zoom-in view of the area of node scales from 100 to 10k; **b** Line-chart visualization of time cost results of all the libraries with the edge-to-node ratios of 1–10. The *x*-axis denotes the edge-to-node, *y*-axis denotes the time cost, a colored line represents a library, and a point on a line represents the weighted average time cost of visualizing the 19 graph datasets (corresponding to the 19 node scales from 100 to 10k) at an edge-to-node ratio

**Fig. 3:**
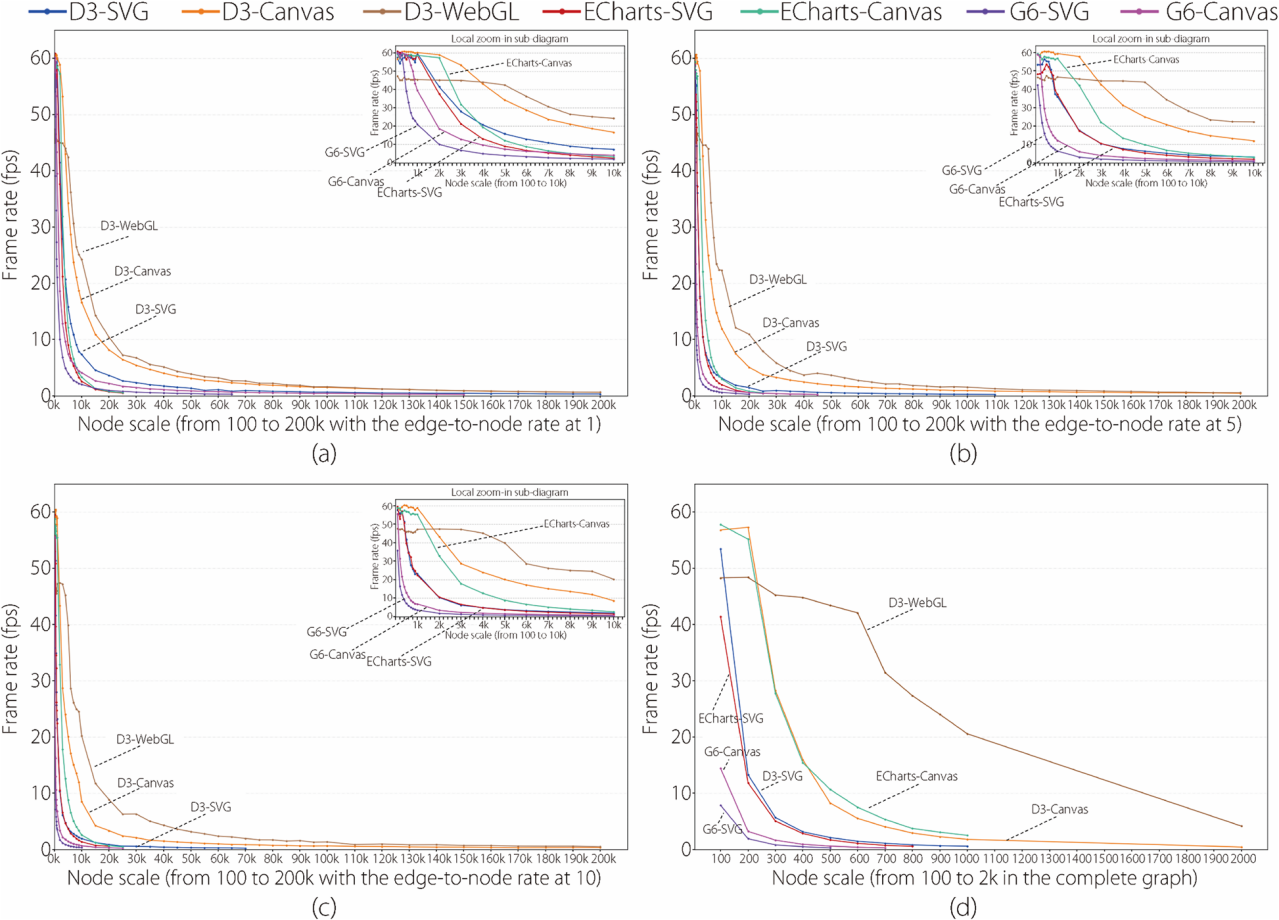
Line-chart visualizations of frame rate results of all the libraries with all the node scales and edge-to-node ratios of 1 (**a**), 5 (**b**), 10 (**c**), and CG (**d**). In each line-chart diagram, the *x*-axis denotes the node scale, the *y*-axis denotes the frame rate, a colored line represents a library, a point on a line represents the frame rate of visualizing a dataset using a library, and the top-right sub-diagram is the zoom-in view of the area of node scales from 100 to 10k

**Fig. 4:**
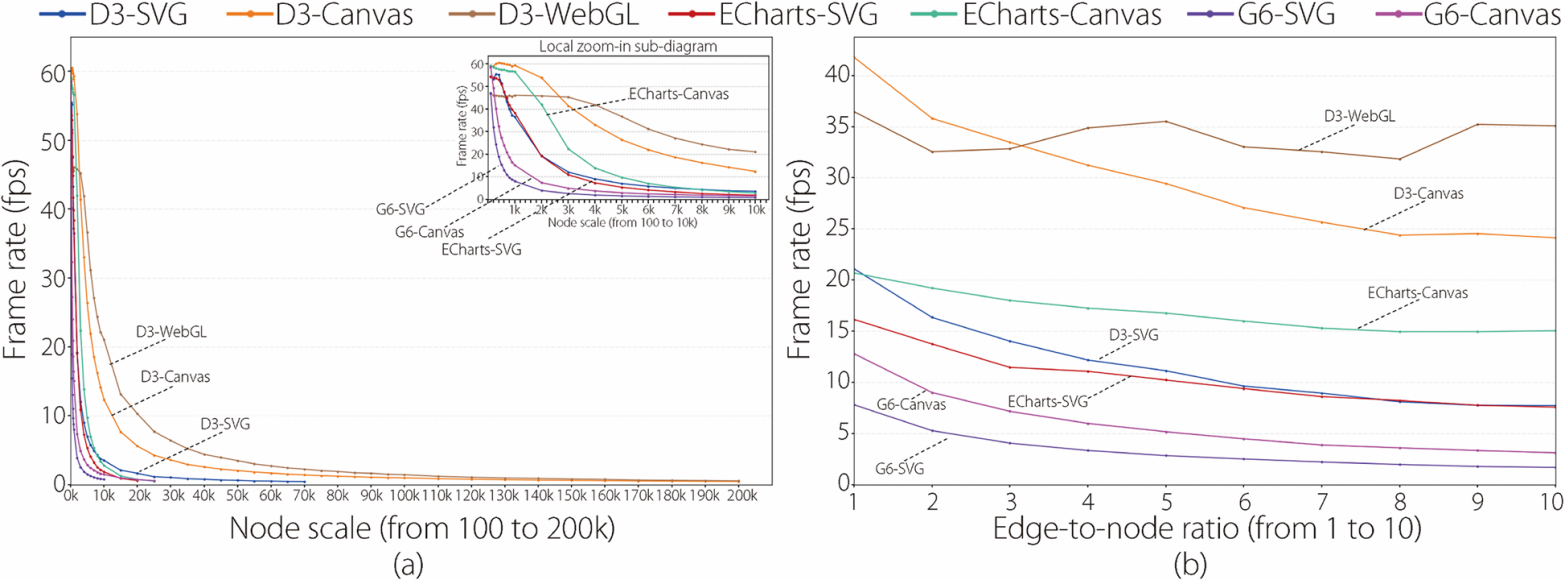
**a** Line-chart visualization of frame rate results of all the libraries with the node scales from 100 to 200k. The *x*-axis denotes the node scale, the *y*-axis denotes the frame rate, a colored line represents a library, a point on a line represents the average frame rate of visualizing the ten graph datasets (corresponding to the ten edge-to-node ratios from 1 to 10, respectively) at a node scale, and the top-right sub-diagram is the zoom-in view of the area of node scales from 100 to 10k; **b** Line-chart visualization of frame rate results of all the libraries with the edge-to-node ratios from 1 to 10. The *x*-axis denotes the edge-to-node, the *y*-axis denotes the frame rate, a colored line represents a library, and a point on a line represents the weighted average frame rate of visualizing the 19 graph datasets (corresponding to the 10 node scales from 100 to 10k) at an edge-to-node ratio

The results of the statistical analysis are presented in various line-chart diagrams, as shown in Figs. 1, 2, 3 and 4.

Figure 1 has four line-chart diagrams. Each diagram was drawn using the time cost results of all the libraries with all the node scales and a specific edge-to-node ratio. The edge-to-node ratios of the four diagrams were set to 1, 5, 10, and CG, respectively. The four edge-to-node ratios were selected for two reasons. First, the four edge-to-node ratios can represent typical low, mid, high, and CG graph densities. They frequently occur in four common network types: scale-free, small-world, dense, and complete. Second, this selection can help shorten the duration of the study. The time cost results for the other edge-to-node ratios are provided in the Supplementary Material. For instance, the blue-colored lines in Fig. 1a and b depict the changing trends of time costs of using the D3-SVG library to visualize the graph datasets when the node scales of the datasets increase from 100 to 200k and the edge-to-node ratios are fixed at 1 and 5. The two lines were drawn using the time costs recorded in the first and fifth columns of Table 1. Notably, the blue D3-SVG line in Fig. 1a starts at a minimum node scale of 100 and ends at a maximum node scale of 200k. The blue D3-SVG line in Fig. 1b starts at the node scale 100 but ends at 110k because ‘null’ values occur in the fifth column of Table 1 when the node scales exceed 110k, indicating that the D3-SVG library cannot visualize datasets larger than 110k nodes within 15 minutes at the edge-to-node ratio of 5 in the experiment.

Figure 2 has two line-chart diagrams. Figure 2a was drawn using the time cost results of all libraries with all the node scales by considering all edge-to-node ratios (excluding CG) at the node scale as a whole. For example, a point on the blue line in Fig. 2a represents the average value of the 10 time costs recorded in a row of Table 1, reflecting the D3-SVG’s average time cost for visualizing the ten graph datasets with the edge-to-node ratios from 1 to 10 at a specific node scale. The blue line shows the overall trend in time costs for using the D3-SVG library to visualize graph datasets as the node scales increase from 100 to 200k, with each node scale incorporating all 10 edge-to-node ratios. The blue D3-SVG line starts at a node scale of 100 but ends at 70k because ‘null’ values occur in Table 1 when the node scales exceed 70k. Thus, the authors cannot accurately assess the overall time performance of the D3-SVG library at any node scale exceeding 70k by considering the edge-to-node ratios from 1 to 10.

Figure 2b was drawn using the time cost results of all libraries with all edge-to-node ratios (excluding CG) by considering node scales from 100 to 10k as a whole. For instance, the blue line in Fig. 2b depicts the changing trend of the time costs of the D3-SVG library when the edge-to-node ratios increased from 1 to 10, and each edge-to-node ratio involved node scales ranging from 100 to 10k. A point on the line denotes the weighted average value of the first 19 time costs recorded in a column of Table 1. The selected node-scale range and the weighted average value computation were carefully considered to generate the figure. The node scale range from 100 to 10k was chosen to facilitate a fair comparison because all the libraries obtained valid time cost results (i.e., no ‘null’ values) in this range. The weighted average value computation was owing to the uneven intervals of the selected node scale range. When generating the experimental datasets, the interval of the node scales from 100 to 1k was set to 100, and the interval of the node scales from 1k to 10k was set to 1k. Thus, when calculating that the 10 time costs the obtained by the node scales from 100 to 1k had a weight of 0.01 and the nine time costs obtained by the node scales from 2k to 10k had a weight of 0.1 when calculating the average value of the first 19 time costs recorded in a column of Table 1.

Figure 3 has four line-chart diagrams drawn using the manner similar to the four diagrams in Fig. 1. The main difference is that the *y*-axis represents the frame rate in Fig. 3 but the time cost in Fig. 1. That is, the four diagrams in Fig. 4 were drawn based on the frame rate results in Table 2. Additional frame rate results are provided in the Supplementary Material. Similarly, Fig. 4 has two line-chart diagrams drawn in a manner similar to the two diagrams in Fig. 2. The main difference is that the *y*-axis represents the frame rate in Fig. 4 but the time cost in Fig. 2.

## Results

### Time cost result analysis

This subsection analyzes the time cost results of the seven experimental library entries with an increase in the node scales and edge-to-node ratios based on Figs. 1 and 2.

Figure 1 has four line-chart diagrams that present the time cost results at the edge-to-node ratios of 1, 5, 10, and CG, respectively. Figure 1a uses seven colored lines to depict the time cost changing trends of the seven experimental libraries as the node scale increases at an edge-to-node ratio of 1. The following three main findings were obtained. (1) The lines begin at the same node scale of 100 but end at different node scales, including ECharts-SVG at 25k, ECharts-Canvas at 25k, G6-SVG at 65k, G6-Canvas at 150k, D3-SVG at 200k, D3-Canvas at 200k, and D3-WebGL at 200k. This indicates that the maximum node scale at which each library could obtain graph visualizations within 15 min differs at the edge-to-node ratio of 1. (2) The lines exhibit approximately linear growth trends with different slopes. The D3-SVG, D3-Canvas, and D3-WebGL lines present gentle slopes, indicating slow growth in time costs. G6-SVG and G6-Canvas present medium slopes, indicating moderate growth in time costs, whereas ECharts-SVG and ECharts-Canvas present steep slopes, indicating the rapid growths of time costs. (3) The lines align closely within node scales from 100 to 3k; however, they diverge rapidly starting from the node scale of 3k, as depicted in the local zoom-in view at the top right of Fig. 1a. This phenomenon indicates that a node scale of 3k is the turning point. The time costs of the libraries are close before this point but are distinguished after it.

These findings could be attributed to three main factors. (Factor 1) Graph layout algorithms. D3, ECharts, and G6 libraries use different variants of force-directed layout algorithms, resulting in different computational time costs for graph layout generations. According to the official documentations of the libraries, D3 uses the Barnes-Hut method [53] to accelerate the computational process of repulsive forces; however, ECharts and G6 do not mention such an optimization. Therefore, D3 generally exhibited a better time performance than ECharts and G6 in the graph layout algorithms. (Factor 2) Graph rendering mechanisms. SVG, Canvas, and WebGL differ primarily in terms of their rendering mechanisms. SVG, a vector graphic-based rendering mechanism, relies on the document object model (DOM) to convert graphic elements into pixels, leading to relatively long rendering times owing to DOM maintenance [35]. Canvas, a resolution-dependent bitmap rendering mechanism, directly renders graphics at fixed pixel resolutions and provides moderate rendering times by avoiding DOM maintenance [36]. WebGL, an evolution of Canvas, uses a GPU to manage the rendering pipeline and process the rendering tasks in parallel, with relatively short rendering times [24]. (Factor 3) Library building methods. D3 is a low-level encapsulated library [10] that allows the direct definition of graphic element properties, styles, and transitions, presenting a short workflow for controlling graph elements. ECharts and G6 are high-level encapsulated libraries [11, 12] that use built-in functions and input parameters to define graphical elements. Such high-level encapsulation reduces programming complexity; however, it leads to a long inner-library workflow. Therefore, generating node-link visualizations using D3 generally requires less time.

The authors further analyze the results presented in Fig. 1a based on the three factors. D3-SVG exhibits shorter time costs than G6-SVG and ECharts-SVG, and D3-Canvas exhibits shorter time costs than G6-Canvas and ECharts-Canvas, as indicated by the gentle slopes and large ending node scales of D3-SVG and D3-Canvas. This is mainly because D3 adopts a force computation optimization and a low-level encapsulation method, as mentioned in Factors 1 and 3. D3-Canvas and G6-Canvas have shorter time costs than D3-SVG and G6-SVG. This result can be explained by Factor 2: Canvas generally requires shorter rendering times than SVG; however, ECharts-SVG and ECharts-Canvas have close time costs because ECharts uses virtual DOM to accelerate SVG rendering. Moreover, D3-Canvas has slightly shorter time costs than D3-WebGL; however, Factor 2 indicates that WebGL commonly presents a better time performance than Canvas. This is because the time performance advantage of WebGL is not easily apparent when only a few graphic elements for rendering are available at a small edge-to-node ratio. This advantage is evident in the time cost results at high edge-to-node ratios, as shown in Fig. 1b, c, and d.

Figure 1b and c show the time cost results of the seven experimental libraries at edge-to-node ratios of 5 and 10, respectively. The three findings presented in Fig. 1a still exist; however, a few new details can be found in the comparison. (1) The lines of ECharts-SVG, G6-SVG, G6-Canvas, and D3-SVG end at smaller node scales. (2) The line slopes of the libraries increase to different extents. (3) The turning point for the time costs of libraries changes from 3k to 1k nodes. All the new details are owing to the higher edge-to-node ratios. The number of edges increases significantly, resulting in more time costs in generating graph layouts and rendering graphic elements.

Figure 1d shows the time cost results of the seven experimental libraries at the edge-to-node ratio of CG. The extremely increased number of edges increases the time performance differences of the libraries. The slopes of the seven lines are clearly distinguishable. D3-WebGL presents the best time cost result with a superior force computation optimization and a low-level encapsulation from D3 and a higher performance graph rendering mechanism from WebGL. G6-SVG presents the worst time cost result with inferior force computation optimization, high-level encapsulation from G6, and lower performance graph rendering mechanisms from SVG.

Figure 2a uses seven colored lines to depict the overall changing trends of the time cost for the seven experimental libraries as the node scale increases, incorporating edge-to-node ratios from 1 to 10 as a whole. The lines start at the same node scale of 100 but end at different node scales and show approximately linear growth with different slopes. A line with a slower slope and a larger end node scale indicates better time cost performance for the corresponding library. The comparative results are as follows: D3-WebGL > D3-Canvas > D3-SVG > G6-Canvas > ECharts-Canvas $\approx$ ECharts-SVG > G6-SVG.

Figure 2b uses seven colored lines to depict the overall changing trends in time cost for the seven experimental libraries as the edge-to-node ratio increases, incorporating node scales from 100 to 10k nodes. The seven lines exhibit approximately linear growth trends with different slopes. A line with a slower slope indicates better time cost performance for the corresponding library as the edge-to-node ratio increases. The comparative results are as follows: D3-WebGL $\approx$ D3-Canvas > D3-SVG > ECharts-Canvas > G6-Canvas $\approx$ ECharts-SVG > G6-SVG.

### Frame rate results analysis

This subsection analyzes the frame rate results of the seven experimental library entries with increasing node scales and edge-to-node ratios based on Figs. 3 and 4.

Figure 3 has four line-chart diagrams that present the frame rate results of the seven experimental libraries at the edge-to-node ratios of 1, 5, 10, and CG, respectively. For analytical purposes, the authors stipulate that a frame rate exceeding or below 30 fps is called a high or low frame rate, because 30 fps is the minimum frame rate necessary for smooth human-vision perception [20].

Figure 3a uses seven colored lines to depict the changing trends in the frame rates of the experimental libraries as the node scale increases at an edge-to-node ratio of 1. Two main findings are presented in Fig. 3a. First, the lines present different changing trends in different node scale ranges. At small node scales, all the lines maintain high frame rates. After reaching certain node scales, the lines of D3-Canvas and D3-WebGL exhibit gentle declines, whereas those of the other five libraries exhibit rapid declines. As the node scale increases further, all the lines decline slowly, approaching zero fps. Second, each line has a different maximum node scale for obtaining high frame rates called HFR-MaxNS in the latter. The HFR-MaxNSs of G6-SVG, G6-Canvas, ECharts-SVG, D3-SVG, ECharts-Canvas, D3-Canvas, and D3-WebGL are 600, 1k, 2k, 2k, 3k, 5k, and 7k, respectively.

The two main findings can be explained by the factors summarized in Fig. 1a. D3 adopts a force computation optimization (Factor 1) and a low-level encapsulation (Factor 3). WebGL and Canvas adopt higher-performance graph rendering mechanisms than SVG (Factor 2). Therefore, D3-WebGL and D3-Canvas present the best frame rate results, as indicated by the slowest declines and maximum HFR-MaxNSs of the D3-WebGL and D3-Canvas lines compared with the other five lines in Fig. 3a. Additionally, the authors observe in Fig. 3a that D3-SVG obtains better frame rate results than G6-SVG and ECharts-SVG, G6-Canvas has better frame rate results than G6-SVG, and ECharts-Canvas has better frame rate results than ECharts-SVG.

Two interesting phenomena were observed. (1) The frame rate of D3-WebGL (50 fps) was lower than that of the other five libraries (60 fps) at a minimum examined node scale of 100. The initialization process of WebGL includes two special operations: shader compilation and buffer management, which lowers the frame rate at the initialization stage. (2) The frame rate results of the two ECharts libraries are superior to those of the two G6 libraries; however, the time cost results of the two ECharts libraries are inferior to those of the two G6 libraries. This is mainly because ECharts supports a progressive visualization mechanism to ensure the rendering of a limited number of graphic elements in each frame.

Figure 3b, c, and d show the frame rate results of the seven experimental libraries as the node scale increases at edge-to-node ratios of 5, 10, and CG, respectively. The two main findings obtained in Fig. 3a still exist; however, new details can be found in the comparison. (1) The lines in the three figures decline more rapidly than the lines in Fig. 3a. (2) The HFR-MaxNS of each library decreases. In Fig. 3b, c, and d, the HFR-MaxNSs of D3-WebGL are at 6k, 5k, and 700, respectively; those of D3-Canvas are at 4k, 2k, and 200, respectively; those of D3-SVG are at 1k, 600, and 100, respectively; those of ECharts-Canvas are at 2k, 2k, and 200, respectively; and those of ECharts-SVG are at 1k, 700, and 100, respectively. In Fig. 3b and c, the HFR-MaxNSs of G6-SVG are 200 and 100, respectively, and 300 and 200, respectively, for G6-Canvas. However, as shown in Fig. 3d, G6-SVG and G6-Canvas did not achieve high frame rates at the minimum node scale examined in the experiment. These new features are related to an increase in the edge-to-node ratio. A large increase in the number of edges deteriorates the rendering performance of all the libraries. Moreover, the rendering advantage of WebGL using GPU’s parallel processes becomes increasingly apparent as the edge-to-node ratio increases. Thus, at edge-to-node ratios of 5, 10, and CG, the rendering performance of D3-WebGL is superior to that of the other libraries.

Figure 4a uses seven colored lines to depict the overall changing trends of the frame rate for the seven libraries as the node scale increases, incorporating edge-to-node ratios from 1 to 10. The lines present approximately nonlinear declining trends at different speeds and end at different node scales. A line that declines more slowly and ends at a larger node scale indicates a better frame rate performance for the corresponding library as the node scale increases. The comparative results are as follows: D3-WebGL > D3-Canvas > ECharts-Canvas > ECharts-SVG $\approx$ D3-SVG > G6-Canvas > G6-SVG.

Figure 4b uses seven colored lines to depict the overall changing trends in the frame rate for the seven experimental libraries as the edge-to-node ratio increases, incorporating node scales from 100 to 10k nodes. The seven lines exhibited approximately linear declining trends with different slopes. A line with a slower slope indicates better frame rate performance for the corresponding library as the edge-to-node ratio increases. The comparative results are as follows: D3-WebGL > D3-Canvas > ECharts-Canvas > ECharts-SVG > D3-SVG > G6-Canvas > G6-SVG.

### Application-oriented guidance and usage cases

The previous two subsections offer an in-depth analysis of the time cost and frame rate results. However, these analyses are not user-friendly in providing application-oriented guidance to end users when selecting a web-based graph visualization library based on efficiency requirements. The experimental results are summarized in Tables 3 and 4. The authors also provide three usage cases that incorporate the three main factors end users considered when choosing libraries to demonstrate how to apply the results in the tables.

**Table 3:** Application-oriented time cost results

Time limit (min)	Edge-to-node ratio	D3-SVG	D3-Canvas	D3-WebGl	ECharts-Canvas	ECharts-SVG	G6-Canvas	G6-SVG
15	1	200k	200k	200k	25k	25k	150k	65k
	2	180k	200k	200k	25k	25k	95k	40k
	3	140k	200k	200k	25k	25k	70k	30k
	4	130k	200k	200k	25k	20k	55k	25k
	5	110k	200k	200k	25k	20k	45k	20k
	6	100k	200k	200k	25k	20k	40k	20k
	7	85k	200k	200k	25k	20k	30k	15k
	8	75k	200k	200k	25k	20k	30k	15k
	9	70k	200k	200k	25k	20k	25k	10k
	10	70k	200k	200k	25k	20k	25k	10k
5	1	90k	200k	180k	10k	10k	60k	25k
	2	65k	180k	180k	15k	10k	30k	15k
	3	50k	160k	180k	10k	10k	20k	10k
	4	45k	140k	180k	15k	10k	15k	10k
	5	40k	130k	170k	15k	10k	15k	8k
	6	35k	110k	170k	10k	10k	10k	7k
	7	25k	100k	160k	10k	10k	10k	6k
	8	25k	100k	160k	10k	10k	10k	5k
	9	20k	85k	150k	10k	10k	9k	5k
	10	20k	85k	150k	10k	9k	9k	4k
1	1	20k	50k	55k	6k	6k	10k	5k
	2	10k	45k	55k	6k	5k	6k	3k
	3	10k	30k	50k	6k	4k	5k	2k
	4	10k	30k	50k	6k	4k	4k	2k
	5	9k	25k	50k	6k	4k	3k	1k
	6	7k	20k	50k	5k	3k	2k	1k
	7	6k	20k	50k	5k	3k	2k	1k
	8	6k	20k	45k	5k	3k	2k	1k
	9	5k	20k	45k	5k	2k	2k	1k
	10	5k	15k	45k	5k	2k	1k	1k

The row represents the time limit (15, 5, or 1 min) and the edge-to-node ratio (ranging from 1 to 10). The column of the cell represents the library. A cell corresponds to the maximum node scale of a graph dataset that can be visualized within a specific time limit and at a specific edge-to-node ratio

Table 3 summarizes the maximum node scales of graph datasets on which each experimental library can generate node-link visualizations within three given time limits (i.e., 15-, 5-, and 1-min) at edge-to-node ratios ranging from 1 to 10. The three-time limits correspond to the maximum acceptable, common, and optimal time costs, respectively. Table 3 serves as a quick reference of the time cost results obtained in the experiment for practical use. For example, the cell in the first row and first column indicates that D3-SVG visualizes a graph dataset with an edge-to-node ratio of 1. Within 15 min, the maximum node scale of the graph dataset is 200k nodes. The cell in the 20th row and 7th column indicates that the maximum node scale that can be visualized using G6-SVG within 5 min at an edge-to-node ratio of 10 is 4k nodes.

Table 4 summarizes the maximum node scales of graph datasets on which each experimental library can generate node-link visualizations while maintaining a frame rate above 30 fps or 10 fps at edge-to-node ratios ranging from 1 to 10. Thirty fps is the minimum frame rate necessary for smooth human vision perception, whereas 10 fps is the empirical threshold below which human eyes can detect stuttering in animations. Table 4 serves as a quick reference of the frame rate results obtained in the experiment for practical use. For example, the cell in the first row and column indicates that when using D3-SVG to visualize a graph dataset with an edge-to-node ratio of 1 and a frame rate above 30 fps, the maximum node scale of the graph dataset is 2k nodes. The cell in the 20th row and 7th column indicates that the maximum node scale that can be visualized using G6-SVG with a frame rate above 10 fps at an edge-to-node ratio of 10 is 300 nodes.

Three usage cases are considered to demonstrate how to use the results in Tables 3 and 4.

**Table 4:** Application-oriented frame rate results

Frame rate (fps)	Edge-to-node ratio	D3-SVG	D3-Canvas	D3-WebGl	ECharts-Canvas	ECharts-SVG	G6-Canvas	G6-SVG
30	1	2k	5k	7k	3k	2k	1k	600
	2	1k	5k	6k	2k	1k	800	300
	3	1k	5k	5k	2k	1k	600	200
	4	1k	4k	6k	2k	1k	300	200
	5	1k	4k	6k	2k	1k	300	200
	6	1k	3k	6k	2k	1k	300	100
	7	800	3k	5k	2k	900	200	100
	8	700	3k	5k	2k	800	200	100
	9	600	2k	6k	2k	700	200	100
	10	600	2k	5k	2k	700	200	100
10	1	7k	15k	20k	5k	4k	3k	2k
	2	5k	15k	20k	5k	4k	2k	1k
	3	4k	10k	20k	5k	3k	1k	900
	4	3k	10k	20k	4k	3k	1k	700
	5	3k	10k	20k	4k	3k	1k	600
	6	2k	10k	20k	4k	2k	1k	500
	7	2k	9k	20k	4k	2k	800	400
	8	2k	9k	15k	4k	2k	700	400
	9	2k	8k	15k	4k	2k	700	300
	10	2k	9k	15k	4k	2k	600	300

The row represents the frame rate threshold (30 or 10 fps) and the edge-to-node ratio (ranging from 1 to 10). The column of the cell represents the library. A cell corresponds to the maximum node scale of a graph dataset that can be visualized above a specific frame rate threshold and at a specific edge-to-node ratio

Alan, a front-end engineer, is developing a web application for social network data analysis. The web application requires the generation of node-link visualizations using social network data with less than 2k nodes and an edge-to-node ratio of approximately 1. End users of the web application may interact with node-link diagrams through actions such as dragging and selecting nodes, which require smooth animation. Therefore, Alan’s efficiency requirements can be summarized as generating node-link visualizations of social network data with 2k nodes and an edge-to-node ratio of 1 within 1 min while maintaining a frame rate above 30 fps. Additionally, Alan requires a visualization library with low programming complexity to enhance development efficiency.

Considering the efficiency requirements, Alan refers to the 21st row in Table 3. The results in this row indicate that all libraries can visualize graph data with 2k000 nodes and an edge-to-node ratio of 1 within a 1-min limit. Thereafter, Alan refers to the first row in Table 4. The results in this row indicate that D3-SVG, D3-Canvas, D3-WebGL, ECharts-SVG, and ECharts-Canvas can visualize graph data with 2k nodes and an edge-to-node ratio of 1 while maintaining the frame rate above 30 fps. D3-SVG, D3-Canvas, D3-WebGL, ECharts-SVG, and ECharts-Canvas satisfies Alan’s efficiency requirements by intersecting the findings from Tables 3 and 4. Additionally, considering the requirement of low programming complexity, Alan prefers ECharts, which offers a high-level encapsulation of node-link visualization generation functions. Therefore, considering both efficiency and programming complexity, ECharts-Canvas and ECharts-SVG are the desired library options for Alan to develop the web applications.

Bob, a product architect, is designing a web application for supply chain data analysis. The application requires the generation of node-link visualization using supply chain network data with a maximum node scale of 5k and an edge-to-node ratio of approximately 5. The application requires avoiding noticeable animation stutters. Therefore, Bob’s efficiency requirements can be summarized as generating node-link visualizations of supply chain graphs with 5k nodes and an edge-to-node ratio of 5 within 1 min while maintaining a frame rate above 10 fps. The results in the 25th row of Table 3 indicate that D3-SVG, D3-Canvas, D3-WebGL, and ECharts-Canvas can visualize graph data with 5k nodes and an edge-to-node ratio of 5 within 1 min. The results in the 15th row of Table 4 indicate that D3-Canvas and D3-WebGL can maintain a frame rate above 10 fps while visualizing graph data with 1k nodes and an edge-to-node ratio of 5. By combining these results, D3-Canvas and D3-WebGL satisfy Bob’s efficiency requirements.

Christy, a big data engineer, is working on a large-scale graph analysis study. The study requires the generation of a node-link visualization of a graph dataset with 100k nodes and an edge-to-node ratio of approximately 10. Christy focuses on whether the visualization could be generated within the maximum acceptable time limit with no specific requirements for the frame rate. Therefore, Christy’s efficiency requirements can be summarized as generating a node-link diagram using a graph dataset with 100k nodes and an edge-to-node ratio of 10 within 15 min. As the results in the 10th row of Table 3 indicated, both D3-Canvas and D3-WebGL satisfy Christy’s efficiency requirements. Considering the need to adopt one of the additional libraries to achieve the WebGL rendering part in D3.js, which has higher programming complexity compared to D3-Canvas. Therefore, Christy prefers D3-Canvas to complete the study.

## Discussion

This section discusses the limitations of this study and proposes directions for future work.

The scales and application scenarios of the graph datasets used in this study have limited coverage. The scales were capped at 200k nodes. Thus, evaluating the performance of web-based graph visualization libraries beyond this scale is worth further investigation. The authors anticipate that such evaluations will likely involve libraries supporting the WebGL rendering method and require advanced acceleration techniques such as WebWorker programming and parallel computing [54, 55]. Additionally, the application scenarios related to the datasets were limited. The potential applications of node-link diagrams are extensive, and include genomic research, neural networks, and infrastructure mapping [56–58]. However, these scenarios were not included in the datasets. The applicability of our findings to these scenarios remains uncertain and warrants further investigation.

The number of graph visualization libraries was also limited. Other libraries available on the market, such as Cytoscape.js, Vis.js, and Sigma.js, were not included [25, 59, 60]. Moreover, although stable versions of D3.js, ECharts.js, and G6.js were selected, these libraries have newly released versions, such as D3.js version 7.9.0, released in March 2024; ECharts.js version 5.5.1, released in June 2024; and G6.js version 5.0.17, released in August 2024. Further explorations that introduce additional libraries and compare the performance differences across different versions of the same library would also be worthwhile.

The experiments in this study were conducted using the default configuration parameters for each library. The parameters of these libraries can be further adjusted using the results of this study. Fine-tuning the parameters may improve performance. The experiments were conducted using specific hardware and software. However, the results may exhibit subtle variations in different experimental environments. Based on our experience, adopting a more advanced GPU and multicore processor with higher clock speeds for hardware, as well as using the latest versions of graph visualization libraries and optimizing browser settings for software, could further enhance the efficiency of the libraries. This warrants further investigation.

The experiment measured the time cost as the duration from loading a dataset into a library to completing 200 iterations of layout computation and rendering. This was chosen because common library users are primarily concerned with the overall time required to visualize a node-link diagram. From a technical perspective, the layout computation and rendering processes involve multiple technical steps, including initializing node positions, calculating forces between nodes, updating node positions, drawing nodes and edges, rasterizing graphical elements into pixels, and compositing the final image. For researchers and library developers, analyzing the time performance of each step individually for experimental libraries could provide insights into identifying performance bottlenecks and facilitate the optimization of the library. This should be further explored in future studies.

This study used NetV.js to implement the WebGL rendering method. However, other WebGL implementations, such as the native WebGL API or Three.js [61], could also be employed, which may result in different performance results that were not addressed in this study. The impact of different WebGL implementations on the rendering performance should be explored in future research.

The experimental results were based on static node-link diagrams, where the number of nodes and edges remained constant. This study does not cover dynamic node-link diagrams, where nodes and edges can be added or removed. The performance of different libraries in dynamic node-link diagrams is an important area of investigation for future research [62].

## Conclusions

This study investigates the node-link graph visualization efficiency of popular web-based libraries. The authors selected seven library entries: D3-SVG, D3-Canvas, D3-WebGL, ECharts-SVG, ECharts-Canvas, G6-SVG, and G6-Canvas, and prepared 481 graph datasets with node scales ranging from 100 to 200k and edge-to-node ratios from 1 to 10 (including CG) to conduct a controlled experiment. They recorded the time costs and frame rates using libraries to visualize the datasets as the experimental results. After a theoretical and empirical analysis of the results, they summarized the overall trends and relative strengths of the efficiency performance of the libraries and reorganized the results and findings into application-oriented guidelines and three usage cases to help end users quickly select the desired libraries based on their specific efficiency requirements of node-link graph visualizations. The authors hope that this study offers application-oriented perspectives on the evolution of graph visualization techniques.

## Supplementary Information

Supplementary Material 1.

## Data Availability

The dataset supporting the conclusions of this article is included within the article and its additional file.

## Abbreviations

API: Application programming interface
fps: Frames per second
SVG: Scalable vector graphics
WebGL: Web graphics library
GPU: Graphics processing unit
CG: Complete graph
DOM: Document object model
HFR-MaxNS: Maximum node scale for high frame rates
