# Supplementary material for: Graph visualization efficiency of popular web-based libraries
Source: Vis Comput Ind Biomed Art. 2025 May 8;8:12. doi: 10.1186/s42492-025-00193-y (PMC12061801; doi:10.1186/s42492-025-00193-y)
Supplement: Supplementary file 1 — Supplementary Material 1. [file 42492_2025_193_MOESM1_ESM.pdf]

Graph Visualization Efficiency of Popular Web-based Libraries

Supplementary Material

1. Experimental Parameter Settings

Supplementary Tables S1, S2, and S3, provide the detail experimental parameters for the D3.js, ECharts.js, and G6.js libraries in this study, respectively. Supplementary Tables S4, S5, and S6 provide specific experimental parameters for the library entries using D3.js, using ECharts.js, and using G6.js in this study, respectively.

Table S1. Detailed experimental parameter settings for the D3.js (5.16.0) library in this study.

Library	Parameter Name	Value	Default or Not
D3.js	simulation.force:charge	d3.forceManyBody()	Not Default
	simulation.force:center	d3.forceCenter(1500,1500)	Not Default
	simulation.alpha	1	Default
	simulation.alphaMin	0.001	Default
	simulation.alphaDecay	0.0228	Default
	simulation.alphaTarget	0	Default
	simulation.velocityDecay	0.4	Default
	manyBody.strength	-30	Default
	manyBody.theta	0.9	Default
	manyBody.distangceMin	1	Default
	manyBody.distangceMax	infinity	Default
	d3.forceX()	0	Default
	d3.forceY()	0	Default

Table S2. Detailed experimental parameter settings for the ECharts.js (5.4.2) library in this study.

Library	Parameter name	Value	Default or Not
Echarts.js	type	graph	Not Default
	center	['50%','50%']	Not Default
	layout	force	Not Default
	force.initLayout	null	Default
	force.repulsion	50	Default
	force.gravity	0.1	Default
	force.edgeLength	30	Default
	force.layoutAnimation	true	Default
	force.friction	0.6	Default

Table S3. Detail experimental parameter settings for the G6.js (4.8.7) library in this study.

Library	Parameter name	Value	Default or Not
G6.js	type	force	Not Default
	center	[1500,1500]	Not Default
	linkDistance	50	Default
	nodeStrength	null	Default
	edegStrength	null	Default
	preventOverlap	false	Default
	colideStrength	1	Default
	nodeSize	10	Default
	forceSimulation	null	Default
	alpha	0.3	Default

	alphaDecay	0.028	Default
	alphaMin	0.001	Default
	clustering	false	Default
	workerEnabled	false	Default

Table S4. Specific experimental parameter settings for the library entries using D3.js library in this study.

Library Entry	Node-size	Line-width	Renderer
**D3-SVG**	r:0.5	stroke-width:1	SVG
**D3-Canvas**	arc.radius:0.5	lineWidth:1	Canvas
**D3-WebGL**	node.r:0.5	link.strokeWidth:1	WebGL

Table S5. Specific experimental parameter settings for the library entries using ECharts.js library in this study.

Library Entry	Node-size	Line-width	Renderer
**ECharts-SVG**	series-graph.symbolSize:1	series-graph.lineStyle. width:1	SVG
**ECharts -Canvas**			Canvas

Table S6. Specific experimental parameter settings for the library entries using G6.js library in this study.

Library Entry	Node size	Line width	Renderer
**G6-SVG**	defaultNode.size:1	defaultEdge.linewidth:1	SVG
**G6-Canvas**			Canvas

2. Time Cost Results

This section introduces the time cost results of visualizing the 481 graph datasets (47 node scale levels × 10 edge-to-node ratios + 11 node-scale levels × 1 special edge-to-node ratio of complete graph) using the library entries in this paper. Supplementary Tables S7, S8, S9, S10, S11, and S12 correspond to the time cost results of visualizing the 481 graph datasets using the D3-Canvas, D3-WebGL, ECharts-Canvas, ECharts-SVG, G6-Canvas and G6-SVG libraries, respectively. A cell corresponds to the average time cost of visualizing a graph dataset three times. The row and column of the cell represent the node scale and edge-to-node ratio of the dataset, respectively. CG represents complete graph. The cells exceeding 2,000 nodes in the CG column were set with gray background. The time cost results of visualizing the 481 datasets using the D3-SVG library are provided in Table 1 of the paper.

Table S7. Time cost results of visualizing the 481 datasets using the D3-Canvas library.

**Time cost (s)**		**Edge-to-node ratio**										
		**1**	**2**	**3**	**4**	**5**	**6**	**7**	**8**	**9**	**10**	**CG**
**Node scale**	**100**	3.4	3.5	3.3	3.3	3.3	3.3	3.3	3.3	3.3	3.4	3.4
	**200**	3.3	3.3	3.3	3.3	3.3	3.3	3.3	3.3	3.3	3.4	3.5
	**300**	3.3	3.3	3.3	3.3	3.3	3.3	3.3	3.3	3.3	3.3	7.0
	**400**	3.3	3.3	3.3	3.3	3.3	3.3	3.3	3.3	3.3	3.3	12.5
	**500**	3.3	3.3	3.3	3.3	3.3	3.3	3.3	3.3	3.3	3.3	24.4
	**600**	3.3	3.3	3.3	3.3	3.3	3.3	3.3	3.3	3.3	3.3	36.7
	**700**	3.3	3.3	3.3	3.3	3.3	3.3	3.3	3.3	3.3	3.3	50.4
	**800**	3.3	3.3	3.3	3.3	3.3	3.3	3.3	3.4	3.3	3.3	70.1
	**900**	3.3	3.3	3.3	3.3	3.3	3.3	3.4	3.4	3.4	3.4	91.7
	**1k**	3.3	3.3	3.3	3.3	3.3	3.3	3.3	3.3	3.3	3.4	113.6
	**2k**	3.3	3.4	3.4	3.4	3.4	3.4	3.7	3.8	4.4	4.5	463.7
	**3k**	3.4	3.8	3.8	4.3	4.7	5.2	5.8	6.3	6.7	7.0	
	**4k**	3.6	4.6	5.1	5.8	6.4	7.1	7.7	8.2	8.0	8.3	
	**5k**	4.5	5.8	6.6	7.3	8.0	8.8	9.8	10.5	9.6	10.0	
	**6k**	5.4	7.0	8.0	8.8	9.6	11.0	11.7	12.7	11.3	11.7	
	**7k**	6.4	8.4	9.4	10.4	11.7	12.9	13.7	15.3	13.0	13.3	
	**8k**	7.4	9.5	11.0	12.3	13.6	14.9	15.9	17.0	14.4	14.7	
	**9k**	8.2	10.8	12.5	13.8	15.1	16.7	17.8	19.5	20.7	16.8	

Time cost (s)		Edge-to-node ratio										
		**1**	**2**	**3**	**4**	**5**	**6**	**7**	**8**	**9**	**10**	**CG**
**Node scale**	**10k**	9.5	12.0	13.6	15.7	16.8	18.6	20.2	21.5	23.3	24.0	
	**15k**	14.1	18.3	21.3	24.3	26.7	28.8	36.8	37.5	41.5	47.9	
	**20k**	18.7	24.6	28.7	31.5	40.1	41.7	52.5	55.6	59.4	60.6	
	**25k**	24.3	31.4	36.6	44.3	53.7	60.1	64.1	68.8	77.3	84.2	
	**30k**	29.9	37.2	43.7	52.3	62.3	69.4	77.6	89.8	95.4	97.6	
	**35k**	36.0	43.5	60.5	67.2	72.7	84.4	92.5	105.3	109.0	123.6	
	**40k**	42.8	50.2	62.3	74.6	82.2	98.4	107.7	117.8	134.9	137.0	
	**45k**	48.8	58.4	71.4	85.1	96.3	108.7	120.9	141.5	150.9	155.9	
	**50k**	53.1	65.3	79.2	92.6	106.9	123.4	137.2	151.4	164.4	174.3	
	**55k**	60.4	73.7	92.4	103.7	119.1	135.5	152.3	165.9	187.0	190.8	
	**60k**	65.2	78.1	97.5	109.1	129.6	147.6	163.0	181.8	201.9	210.1	
	**65k**	72.4	85.7	109.1	122.7	144.3	164.1	176.9	199.0	217.6	230.7	
	**70k**	78.0	93.0	118.8	131.5	152.0	170.7	187.7	211.3	234.6	246.3	
	**75k**	85.3	103.2	127.9	146.2	163.7	185.1	207.9	228.0	253.6	258.5	
	**80k**	91.0	109.3	136.0	156.2	173.0	195.7	218.9	239.0	267.2	280.3	
	**85k**	99.2	117.3	146.3	169.1	185.8	209.1	233.4	257.7	284.0	297.2	
	**90k**	106.7	124.0	155.4	173.9	200.6	217.7	245.5	273.9	300.7	312.7	
	**95k**	115.1	133.5	165.2	183.1	214.6	234.1	256.4	289.6	314.4	331.4	
	**100k**	122.4	138.3	170.8	202.2	219.9	246.4	268.1	296.2	325.7	350.6	
	**110k**	138.6	155.3	190.2	225.4	244.3	272.3	302.0	331.8	357.2	386.5	
	**120k**	155.0	172.5	206.3	247.9	267.0	301.6	329.5	362.6	396.7	420.7	
	**130k**	170.5	188.9	225.8	272.0	291.0	323.5	362.5	391.4	418.9	451.0	
	**140k**	193.0	206.8	243.6	296.2	316.5	353.3	391.7	427.9	459.2	490.9	
	**150k**	207.3	227.8	265.4	323.4	345.6	382.5	421.3	458.6	493.0	528.1	
	**160k**	223.1	249.6	287.6	349.0	369.7	409.9	447.1	489.9	524.5	569.9	
	**170k**	241.1	269.0	308.8	355.1	395.8	438.8	477.0	528.4	569.5	604.6	
	**180k**	257.5	288.0	333.3	386.7	418.3	464.6	507.8	566.1	616.3	638.6	
	**190k**	276.4	310.1	355.8	412.3	441.7	491.9	542.5	595.3	650.8	688.6	
	**200k**	294.5	325.5	372.9	421.1	464.8	526.2	569.2	622.4	667.3	720.7	

Table S8. Time cost results of visualizing the 481 datasets using the D3-WebGL library.

Time cost (s)		Edge-to-node ratio										
		**1**	**2**	**3**	**4**	**5**	**6**	**7**	**8**	**9**	**10**	**CG**
**Node scale**	**100**	3.3	3.3	3.4	3.5	3.3	3.3	3.3	3.3	3.3	3.3	3.3
	**200**	3.3	3.3	3.3	3.3	3.3	3.3	3.3	3.4	3.3	3.3	3.3
	**300**	3.3	3.3	3.3	3.3	3.3	3.3	3.3	3.3	3.3	3.3	3.3
	**400**	3.3	3.3	3.3	3.3	3.3	3.3	3.3	3.3	3.3	3.3	3.3
	**500**	3.3	3.3	3.3	3.3	3.4	3.3	3.3	3.3	3.3	3.3	3.4
	**600**	3.3	3.4	3.3	3.4	3.3	3.3	3.3	3.3	3.4	3.3	3.5
	**700**	3.3	3.3	3.3	3.3	3.3	3.3	3.3	3.3	3.3	3.3	5.3
	**800**	3.3	3.3	3.3	3.3	3.3	3.3	3.3	3.3	3.4	3.3	6.2
	**900**	3.3	3.3	3.3	3.3	3.3	3.3	3.3	3.3	3.3	3.3	7.4
	**1k**	3.3	3.3	3.3	3.3	3.3	3.3	3.3	3.4	3.3	3.3	8.7
	**2k**	3.3	3.3	3.3	3.3	3.3	3.3	3.3	3.4	3.3	3.3	47.6
	**3k**	3.3	3.4	3.4	3.3	3.3	3.4	3.4	3.4	3.4	3.3	
	**4k**	3.5	3.6	3.8	3.5	3.4	3.9	4.1	4.2	3.4	3.4	
	**5k**	3.4	4.8	5.0	4.2	3.6	5.0	4.9	4.8	4.2	4.0	
	**6k**	4.3	5.3	6.0	4.9	4.7	5.1	5.5	6.1	5.0	5.3	
	**7k**	5.3	6.2	7.2	5.9	6.0	5.7	6.7	7.1	5.7	6.2	
	**8k**	6.2	7.3	8.0	7.2	6.6	7.9	6.9	7.1	6.6	6.9	
	**9k**	6.9	7.3	7.6	7.6	7.7	8.7	8.2	8.4	8.9	6.7	
	**10k**	7.5	7.8	7.6	8.2	7.9	8.7	10.0	9.7	8.9	8.6	

Time cost (s)		Edge-to-node ratio										
		**1**	**2**	**3**	**4**	**5**	**6**	**7**	**8**	**9**	**10**	**CG**
**Node scale**	**15k**	12.4	14.5	14.0	12.4	14.4	13.6	13.8	16.3	14.7	15.5	
	**20k**	17.4	16.6	17.3	17.2	17.5	17.7	18.1	18.9	19.3	19.7	
	**25k**	22.6	21.7	23.1	23.7	23.2	23.2	24.4	25.3	25.4	25.9	
	**30k**	26.3	28.3	28.7	28.8	30.0	29.9	29.8	31.9	30.4	30.8	
	**35k**	31.9	32.5	34.3	34.7	36.4	36.9	37.2	39.0	36.8	39.1	
	**40k**	37.8	39.1	40.9	42.0	45.0	44.8	43.8	48.9	45.3	45.4	
	**45k**	44.3	44.7	48.7	50.6	48.8	49.2	52.5	52.2	55.9	54.8	
	**50k**	49.3	52.1	57.1	53.8	54.3	57.8	57.7	61.3	61.8	62.1	
	**55k**	56.7	58.5	64.9	64.3	62.4	65.0	66.0	68.6	69.5	71.4	
	**60k**	62.3	64.5	71.7	67.5	68.3	73.3	75.8	73.1	79.7	82.5	
	**65k**	70.6	72.5	81.1	83.2	80.4	80.9	81.8	82.1	87.2	89.0	
	**70k**	75.4	79.0	89.5	83.5	89.5	91.5	89.0	89.0	94.6	100.7	
	**75k**	85.0	85.9	100.9	91.4	94.8	98.0	97.7	99.4	102.0	105.5	
	**80k**	91.4	85.7	107.2	98.0	110.6	106.5	107.4	102.7	109.6	115.5	
	**85k**	100.1	99.9	124.6	107.9	117.0	114.4	114.0	110.9	121.8	124.3	
	**90k**	107.8	108.3	132.6	120.7	122.3	121.5	122.9	121.0	130.1	134.8	
	**95k**	121.4	118.2	141.5	127.3	127.3	129.1	133.5	137.3	138.1	145.0	
	**100k**	127.5	128.7	132.1	133.2	138.2	132.8	145.9	148.6	150.7	155.7	
	**110k**	138.6	145.8	157.7	163.8	158.0	161.0	174.4	155.6	170.6	177.7	
	**120k**	159.2	168.0	170.7	171.0	176.9	201.2	182.3	184.0	188.6	204.2	
	**130k**	180.8	181.7	188.9	204.5	196.2	217.0	207.2	214.4	221.3	222.8	
	**140k**	205.5	211.4	209.3	224.0	214.3	224.9	233.2	222.7	241.8	242.2	
	**150k**	222.7	223.0	220.3	236.8	238.6	249.3	256.9	251.8	278.4	284.6	
	**160k**	245.5	249.5	244.1	259.0	265.5	270.5	279.5	273.8	301.3	326.8	
	**170k**	264.9	265.6	271.0	274.5	287.0	297.3	309.3	320.3	333.3	343.3	
	**180k**	284.1	286.0	287.2	295.5	306.4	325.7	329.8	344.4	364.2	373.2	
	**190k**	306.1	317.0	314.9	321.4	330.2	354.9	353.9	356.6	402.6	408.1	
	**200k**	324.3	329.0	330.6	351.7	360.0	374.8	400.4	422.8	442.8	462.5	

Table S9. Time cost results of visualizing the 481 datasets using the ECharts-Canvas library.

Time cost (s)		Edge-to-node ratio										
		**1**	**2**	**3**	**4**	**5**	**6**	**7**	**8**	**9**	**10**	**CG**
**Node scale**	**100**	3.5	3.6	3.8	3.6	3.7	3.7	3.6	3.8	3.7	3.7	3.9
	**200**	3.7	3.6	3.7	3.6	3.7	3.7	3.7	3.7	3.8	3.7	7.2
	**300**	3.6	3.7	3.7	3.7	3.8	3.8	3.8	3.9	3.9	4.0	14.7
	**400**	3.8	3.8	3.9	3.9	3.9	4.0	4.1	4.0	4.1	4.0	26.4
	**500**	3.9	4.0	3.9	4.0	4.1	4.1	4.2	4.3	4.4	4.3	38.6
	**600**	4.0	4.1	4.1	4.2	4.2	4.3	4.4	4.5	4.6	4.6	55.6
	**700**	4.1	4.2	4.3	4.3	4.4	4.5	4.7	4.7	4.9	4.9	78.1
	**800**	4.3	4.4	4.5	4.5	4.6	4.7	4.9	5.0	5.3	5.4	112.6
	**900**	4.5	4.6	4.7	4.8	4.8	5.1	5.2	5.3	5.5	5.5	134.6
	**1k**	4.7	4.8	5.0	5.1	5.1	5.3	5.4	5.6	5.7	5.9	165.0
	**2k**	8.3	8.5	8.7	9.0	9.5	10.5	11.7	12.0	12.1	12.1	null
	**3k**	14.3	14.5	16.1	17.9	18.1	19.0	20.7	21.5	22.3	22.5	
	**4k**	21.5	25.2	27.4	28.2	29.9	32.8	32.5	35.5	32.6	31.8	
	**5k**	33.3	36.5	39.6	40.8	41.0	44.3	45.6	46.0	45.8	45.8	
	**6k**	45.9	52.7	55.9	57.5	58.6	61.5	60.8	60.6	62.6	61.0	
	**7k**	61.6	72.4	74.0	74.8	78.4	78.8	80.5	87.3	79.3	79.9	
	**8k**	80.2	89.5	96.7	97.9	94.4	95.8	103.8	107.6	103.5	100.1	
	**9k**	98.9	124.3	113.5	115.9	113.5	124.1	130.0	123.8	126.5	121.0	
	**10k**	122.1	147.7	138.7	135.3	137.7	147.7	146.2	155.9	163.0	156.9	
	**15k**	327.6	295.5	323.1	293.7	288.1	314.1	318.0	323.9	328.3	319.4	

Time cost (s)		Edge-to-node ratio										
		1	2	3	4	5	6	7	8	9	10	CG
Node scale	20k	535.6	504.2	522.7	507.5	523.0	538.5	547.8	531.9	526.9	558.1	
	25k	744.4	742.6	788.7	774.7	790.2	763.9	783.3	793.2	823.0	849.6	
	30k	null	null	null	null	null	null	null	null	null	null	
	35k	null	null	null	null	null	null	null	null	null	null	
	40k	null	null	null	null	null	null	null	null	null	null	
	45k	null	null	null	null	null	null	null	null	null	null	
	50k	null	null	null	null	null	null	null	null	null	null	
	55k	null	null	null	null	null	null	null	null	null	null	
	60k	null	null	null	null	null	null	null	null	null	null	
	65k	null	null	null	null	null	null	null	null	null	null	
	70k	null	null	null	null	null	null	null	null	null	null	
	75k	null	null	null	null	null	null	null	null	null	null	
	80k	null	null	null	null	null	null	null	null	null	null	
	85k	null	null	null	null	null	null	null	null	null	null	
	90k	null	null	null	null	null	null	null	null	null	null	
	95k	null	null	null	null	null	null	null	null	null	null	
	100k	null	null	null	null	null	null	null	null	null	null	
	110k	null	null	null	null	null	null	null	null	null	null	
	120k	null	null	null	null	null	null	null	null	null	null	
	130k	null	null	null	null	null	null	null	null	null	null	
	140k	null	null	null	null	null	null	null	null	null	null	
	150k	null	null	null	null	null	null	null	null	null	null	
	160k	null	null	null	null	null	null	null	null	null	null	
	170k	null	null	null	null	null	null	null	null	null	null	
	180k	null	null	null	null	null	null	null	null	null	null	
	190k	null	null	null	null	null	null	null	null	null	null	
	200k	null	null	null	null	null	null	null	null	null	null	

Table S10. Time cost results of visualizing the 481 datasets using the ECharts-SVG library.

Time cost (s)		Edge-to-node ratio										
		1	2	3	4	5	6	7	8	9	10	CG
Node scale	100	3.5	3.7	3.9	4.0	4.6	4.3	4.9	4.0	4.9	3.8	8.9
	200	3.6	3.7	4.2	4.2	5.4	4.3	6.3	4.4	4.8	5.2	32.9
	300	3.7	3.8	4.8	4.7	5.0	4.5	6.3	7.3	7.4	6.9	77.7
	400	3.9	4.0	4.9	5.5	5.6	6.6	7.7	8.4	8.5	7.7	144.5
	500	4.1	4.8	5.3	5.9	7.2	7.5	7.6	7.7	8.3	10.1	246.6
	600	4.3	5.2	6.0	7.3	7.4	7.9	8.6	10.1	10.8	11.7	399.4
	700	4.5	5.6	6.6	7.6	7.9	9.2	10.2	10.8	11.5	12.6	606.2
	800	4.8	5.5	7.5	7.9	8.3	11.2	11.5	12.8	14.2	15.8	847.6
	900	5.0	6.4	7.4	8.4	10.1	11.7	12.2	13.9	14.5	16.6	null
	1k	5.2	6.9	7.5	9.0	10.7	11.8	14.1	14.8	16.5	17.7	null
	2k	10.5	13.9	16.8	19.5	23.2	25.6	30.9	33.5	36.6	39.2	null
	3k	18.8	24.0	28.9	34.9	38.2	44.9	48.7	51.8	63.0	60.6	
	4k	30.6	37.7	43.1	53.0	55.4	64.4	72.5	74.4	84.0	85.3	
	5k	44.3	50.6	62.4	72.4	76.0	86.7	96.1	102.7	111.8	112.7	
	6k	59.6	69.1	83.9	93.8	99.6	113.4	120.3	132.8	138.6	143.5	
	7k	75.4	91.4	106.7	117.6	126.5	138.4	151.8	160.4	175.4	175.3	
	8k	95.2	123.0	135.0	146.8	151.4	169.0	185.0	212.8	227.0	214.2	
	9k	123.0	145.1	159.3	178.0	186.6	208.4	227.9	243.0	258.9	256.4	
	10k	158.4	178.1	181.7	200.7	212.9	234.7	263.1	277.1	289.8	304.3	
	15k	353.0	368.3	387.2	403.6	412.5	444.5	477.5	532.6	524.1	554.9	
	20k	572.9	575.7	672.2	630.7	687.8	701.2	764.4	784.6	824.3	876.3	

Time cost (s)		Edge-to-node ratio										
		1	2	3	4	5	6	7	8	9	10	CG
Node scale	25k	794.3	826.4	889.8	null	null	null	null	null	null	null	
	30k	null	null	null	null	null	null	null	null	null	null	
	35k	null	null	null	null	null	null	null	null	null	null	
	40k	null	null	null	null	null	null	null	null	null	null	
	45k	null	null	null	null	null	null	null	null	null	null	
	50k	null	null	null	null	null	null	null	null	null	null	
	55k	null	null	null	null	null	null	null	null	null	null	
	60k	null	null	null	null	null	null	null	null	null	null	
	65k	null	null	null	null	null	null	null	null	null	null	
	70k	null	null	null	null	null	null	null	null	null	null	
	75k	null	null	null	null	null	null	null	null	null	null	
	80k	null	null	null	null	null	null	null	null	null	null	
	85k	null	null	null	null	null	null	null	null	null	null	
	90k	null	null	null	null	null	null	null	null	null	null	
	95k	null	null	null	null	null	null	null	null	null	null	
	100k	null	null	null	null	null	null	null	null	null	null	
	110k	null	null	null	null	null	null	null	null	null	null	
	120k	null	null	null	null	null	null	null	null	null	null	
	130k	null	null	null	null	null	null	null	null	null	null	
	140k	null	null	null	null	null	null	null	null	null	null	
	150k	null	null	null	null	null	null	null	null	null	null	
	160k	null	null	null	null	null	null	null	null	null	null	
	170k	null	null	null	null	null	null	null	null	null	null	
	180k	null	null	null	null	null	null	null	null	null	null	
	190k	null	null	null	null	null	null	null	null	null	null	
	200k	null	null	null	null	null	null	null	null	null	null	

Table S11. Time cost results of visualizing the 481 datasets using the G6-Canvas library.

Time cost (s)		Edge-to-node ratio										
		1	2	3	4	5	6	7	8	9	10	CG
Node scale	100	3.4	3.4	3.4	3.4	3.5	3.5	3.5	3.6	3.5	3.7	14.1
	200	3.5	3.5	3.5	3.5	3.6	4.0	4.6	5.0	5.8	6.6	64.6
	300	3.4	3.5	3.6	4.2	5.0	6.0	7.2	7.7	8.7	9.6	123.9
	400	3.5	3.6	5.1	5.9	7.1	8.2	9.9	10.0	11.6	12.7	227.0
	500	3.5	4.3	5.7	7.6	8.9	10.7	12.0	13.0	14.4	15.8	364.4
	600	3.6	5.2	6.6	8.5	10.8	12.1	13.9	15.6	18.0	19.3	529.5
	700	3.9	6.0	7.8	9.9	12.2	14.1	16.0	18.4	20.5	23.3	742.6
	800	4.1	6.7	8.9	11.1	14.0	16.6	19.3	21.6	24.1	26.2	null
	900	4.8	7.5	10.0	13.0	15.2	18.3	21.0	23.7	27.2	29.8	null
	1k	5.2	8.1	11.4	13.9	17.0	19.8	23.2	26.4	28.7	30.1	null
	2k	11.9	16.6	22.3	28.7	34.0	40.9	47.7	50.3	56.9	60.8	null
	3k	16.0	26.1	35.4	42.4	52.7	60.2	68.2	78.8	88.8	94.1	
	4k	21.2	34.4	45.8	57.6	67.8	79.6	96.0	107.2	109.2	115.2	
	5k	27.7	43.9	58.5	71.7	89.4	102.9	115.8	133.3	138.4	144.4	
	6k	33.1	53.5	68.0	86.6	107.9	121.3	142.2	162.8	162.3	173.8	
	7k	35.4	60.5	79.9	101.9	127.3	146.7	169.0	190.2	187.9	204.2	
	8k	42.0	70.6	93.1	117.5	139.9	166.9	194.7	224.0	216.8	230.7	
	9k	46.6	78.2	102.8	135.3	158.3	190.6	223.2	264.7	290.5	253.7	
	10k	49.8	84.3	113.2	143.8	170.2	203.5	245.8	274.5	303.8	316.4	
	15k	78.2	126.6	171.9	236.6	273.6	315.0	361.1	412.3	468.6	490.8	
	20k	93.7	170.3	245.4	304.5	353.3	420.5	510.8	580.5	602.5	661.1	
	25k	117.4	229.0	305.3	383.9	466.3	555.7	630.9	730.7	844.5	874.9	

Time cost (s)		Edge-to-node ratio										
		1	2	3	4	5	6	7	8	9	10	CG
Node scale	30k	141.9	264.6	357.6	470.8	547.0	644.8	756.0	892.1	null	null	
	35k	167.9	319.2	431.2	552.1	665.5	817.0	null	null	null	null	
	40k	181.6	349.0	476.8	613.4	745.1	886.1	null	null	null	null	
	45k	219.6	409.8	565.4	689.1	869.0	null	null	null	null	null	
	50k	229.8	427.0	633.4	765.8	null	null	null	null	null	null	
	55k	264.3	492.4	693.7	880.6	null	null	null	null	null	null	
	60k	278.0	515.3	749.1	null	null	null	null	null	null	null	
	65k	320.5	575.3	816.3	null	null	null	null	null	null	null	
	70k	328.0	599.8	863.2	null	null	null	null	null	null	null	
	75k	372.2	677.4	null	null	null	null	null	null	null	null	
	80k	381.6	716.2	null	null	null	null	null	null	null	null	
	85k	422.7	742.7	null	null	null	null	null	null	null	null	
	90k	436.8	804.1	null	null	null	null	null	null	null	null	
	95k	492.5	868.5	null	null	null	null	null	null	null	null	
	100k	489.0	null	null	null	null	null	null	null	null	null	
	110k	559.0	null	null	null	null	null	null	null	null	null	
	120k	658.0	null	null	null	null	null	null	null	null	null	
	130k	715.9	null	null	null	null	null	null	null	null	null	
	140k	793.7	null	null	null	null	null	null	null	null	null	
	150k	862.6	null	null	null	null	null	null	null	null	null	
	160k	null	null	null	null	null	null	null	null	null	null	
	170k	null	null	null	null	null	null	null	null	null	null	
	180k	null	null	null	null	null	null	null	null	null	null	
	190k	null	null	null	null	null	null	null	null	null	null	
	200k	null	null	null	null	null	null	null	null	null	null	

Table S12. Time cost results of visualizing the 481 datasets using the G6-SVG library.

Time cost (s)		Edge-to-node ratio										
		1	2	3	4	5	6	7	8	9	10	CG
Node scale	100	3.4	3.4	3.8	3.8	4.7	4.2	4.6	5.4	5.7	5.7	26.1
	200	3.4	4.1	4.9	6.2	6.9	8.5	9.5	10.2	11.1	12.6	107.6
	300	3.6	5.4	6.9	8.9	9.5	12.2	13.8	14.3	16.4	17.2	255.6
	400	4.2	6.9	10.1	10.8	12.7	15.3	17.3	19.3	22.0	23.1	460.3
	500	5.3	8.8	10.2	13.8	16.1	19.1	21.7	24.3	27.3	28.7	735.8
	600	6.2	9.6	12.7	15.9	19.3	22.9	25.9	29.1	33.1	35.3	null
	700	7.6	10.8	15.0	19.0	23.0	26.7	30.2	34.3	39.0	41.2	null
	800	8.4	12.8	17.4	21.2	25.4	30.5	34.7	39.5	44.6	47.9	null
	900	9.0	14.4	19.1	24.0	28.2	34.1	38.4	44.8	50.2	53.8	null
	1k	9.8	15.2	20.9	26.3	33.1	37.4	43.8	49.1	54.7	58.2	null
	2k	20.5	31.6	43.5	55.2	67.7	77.7	91.8	100.0	115.2	122.4	null
	3k	30.1	49.5	67.0	85.1	103.0	121.6	136.0	150.9	171.1	182.6	
	4k	42.0	66.3	89.1	113.3	132.0	160.3	181.7	203.2	228.2	243.9	
	5k	52.3	84.3	111.5	142.5	165.5	200.7	231.5	259.6	287.4	308.2	
	6k	62.3	99.6	136.5	173.5	203.3	248.4	276.3	315.4	348.2	373.9	
	7k	76.7	117.3	161.3	205.2	243.9	290.0	325.1	365.7	409.3	439.4	
	8k	85.3	136.7	187.6	237.2	279.8	328.0	368.4	426.9	476.3	512.3	
	9k	94.9	154.6	212.0	269.7	316.8	372.6	419.5	485.5	545.0	585.5	
	10k	105.1	165.9	237.1	296.0	346.5	413.8	473.8	525.7	598.0	623.5	
	15k	162.3	262.3	357.1	447.1	535.4	637.7	720.3	843.7	null	null	
	20k	224.2	354.4	480.1	612.9	753.4	855.2	null	null	null	null	
	25k	284.4	466.1	626.0	782.6	null	null	null	null	null	null	
	30k	351.1	562.3	756.6	null	null	null	null	null	null	null	

Time cost (s)		Edge-to-node ratio										
		1	2	3	4	5	6	7	8	9	10	CG
Node scale	35k	411.8	679.1	null	null	null	null	null	null	null	null	
	40k	478.0	777.6	null	null	null	null	null	null	null	null	
	45k	550.9	null	null	null	null	null	null	null	null	null	
	50k	628.9	null	null	null	null	null	null	null	null	null	
	55k	707.5	null	null	null	null	null	null	null	null	null	
	60k	771.0	null	null	null	null	null	null	null	null	null	
	65k	849.0	null	null	null	null	null	null	null	null	null	
	70k	null	null	null	null	null	null	null	null	null	null	
	75k	null	null	null	null	null	null	null	null	null	null	
	80k	null	null	null	null	null	null	null	null	null	null	
	85k	null	null	null	null	null	null	null	null	null	null	
	90k	null	null	null	null	null	null	null	null	null	null	
	95k	null	null	null	null	null	null	null	null	null	null	
	100k	null	null	null	null	null	null	null	null	null	null	
	110k	null	null	null	null	null	null	null	null	null	null	
	120k	null	null	null	null	null	null	null	null	null	null	
	130k	null	null	null	null	null	null	null	null	null	null	
	140k	null	null	null	null	null	null	null	null	null	null	
	150k	null	null	null	null	null	null	null	null	null	null	
	160k	null	null	null	null	null	null	null	null	null	null	
	170k	null	null	null	null	null	null	null	null	null	null	
	180k	null	null	null	null	null	null	null	null	null	null	
	190k	null	null	null	null	null	null	null	null	null	null	
	200k	null	null	null	null	null	null	null	null	null	null	

3. Frame Rate Results

This section introduces frame rate results of visualizing the 481 graph datasets (47 node scale levels × 10 edge-to-node ratios + 11 node-scale levels × 1 special edge-to-node ratio of complete graph) using the library entries in this paper. Supplementary Tables S13, S14, S15, S16, S17, and S18 corresponds to the frame rate results of visualizing the datasets using the D3-Canvas, D3-WebGL, ECharts-Canvas, ECharts-SVG, G6-Canvas, and G6-SVG library, respectively. A cell corresponds to the average frame rate of visualizing a graph dataset three times. The row and column of the cell represent the node scale and edge-to-node ratio of the dataset, respectively. CG represents complete graph. The cells exceeding 2,000 nodes in the CG column were set with gray background. The frame rate results of visualizing the datasets using the D3-SVG library are provided in Table 2 of the paper.

Table S13. Frame rate results of visualizing the 481 datasets using the D3-Canvas library.

Frame rate (fps)		Edge-to-node ratio										
		1	2	3	4	5	6	7	8	9	10	CG
Node scale	100	56.2	56.5	60.6	60.4	59.2	59.0	58.8	58.6	59.4	59.2	56.8
	200	56.2	59.5	59.8	58.7	58.8	57.7	59.8	58.4	60.2	57.8	57.3
	300	56.2	60.6	60.4	60.3	60.3	59.9	60.1	59.9	60.2	59.4	28.3
	400	56.2	60.8	60.4	60.7	60.6	60.8	60.6	60.3	60.5	60.4	16.0
	500	56.2	60.5	60.8	60.7	60.5	60.5	60.4	59.9	60.1	60.2	8.2
	600	56.2	60.6	60.5	60.4	60.7	60.3	60.3	59.4	59.7	59.1	5.5
	700	56.2	60.6	60.9	60.6	60.1	59.7	58.9	59.1	58.0	59.3	4.0
	800	56.2	60.5	60.6	60.6	60.1	59.0	59.4	58.5	58.5	59.1	2.9
	900	56.2	59.9	60.3	59.7	59.0	59.2	57.4	57.5	58.0	57.8	2.2
	1k	56.2	60.1	59.7	59.6	59.5	59.1	59.1	58.6	58.9	59.0	1.8
	2k	56.2	58.9	58.5	58.2	57.8	56.1	51.8	49.6	45.2	43.3	0.4

Frame rate (fps)		Edge-to-node ratio										
		1	2	3	4	5	6	7	8	9	10	CG
Node scale	3k	56.2	53.2	51.4	46.8	42.6	37.0	34.0	31.7	30.0	28.7	
	4k	56.2	43.0	39.2	34.3	31.3	27.5	25.9	24.7	25.0	24.0	
	5k	56.2	34.2	30.0	27.4	25.0	22.1	20.5	19.1	20.8	20.1	
	6k	56.2	28.7	25.0	22.9	20.8	18.0	17.0	15.7	17.6	17.1	
	7k	56.2	23.7	21.2	19.2	17.1	15.1	14.6	12.9	15.4	15.1	
	8k	56.2	21.1	18.2	16.2	14.8	13.2	12.5	11.7	13.8	13.6	
	9k	56.2	18.6	16.0	14.5	13.3	11.7	11.2	10.3	9.7	11.9	
	10k	56.2	16.6	14.6	12.8	11.9	10.5	9.7	9.2	8.6	8.5	
	15k	56.2	10.9	9.4	8.2	7.5	6.8	5.3	5.4	4.7	4.2	
	20k	56.2	8.2	7.0	6.4	5.0	4.6	3.8	3.5	3.4	3.3	
	25k	56.2	6.4	5.5	4.5	3.7	3.3	3.1	2.9	2.6	2.4	
	30k	56.2	5.4	4.6	3.8	3.2	2.8	2.6	2.2	2.1	2.1	
	35k	56.2	4.7	3.4	3.0	2.8	2.4	2.2	1.9	1.9	1.6	
	40k	56.2	4.0	3.2	2.7	2.4	2.0	1.9	1.7	1.5	1.5	
	45k	56.2	3.4	2.8	2.4	2.1	1.7	1.7	1.4	1.4	1.3	
	50k	56.2	3.1	2.6	2.2	1.9	1.6	1.5	1.3	1.3	1.2	
	55k	56.2	2.8	2.2	1.9	1.7	1.5	1.3	1.2	1.1	1.1	
	60k	56.2	2.6	2.0	1.8	1.6	1.4	1.2	1.1	1.0	1.0	
	65k	56.2	2.3	1.9	1.6	1.4	1.2	1.1	1.0	0.9	0.9	
	70k	56.2	2.1	1.7	1.5	1.3	1.1	1.1	0.9	0.9	0.8	
	75k	2.4	2.0	1.6	1.4	1.3	1.0	1.0	0.9	0.8	0.8	
	80k	2.2	1.8	1.5	1.3	1.2	1.0	0.9	0.9	0.8	0.7	
	85k	2.0	1.7	1.4	1.2	1.1	0.9	0.9	0.8	0.7	0.7	
	90k	1.9	1.6	1.3	1.1	1.0	0.9	0.8	0.7	0.7	0.6	
	95k	1.8	1.5	1.3	1.1	0.9	0.8	0.8	0.7	0.6	0.6	
	100k	1.6	1.5	1.2	1.0	0.9	0.8	0.7	0.7	0.6	0.6	
	110k	1.5	1.3	1.1	0.9	0.8	0.7	0.6	0.6	0.6	0.5	
	120k	1.3	1.2	1.0	0.8	0.8	0.6	0.6	0.5	0.5	0.5	
	130k	1.2	1.1	0.9	0.7	0.7	0.6	0.5	0.5	0.5	0.5	
	140k	1.1	1.0	0.8	0.7	0.6	0.5	0.5	0.5	0.4	0.4	
	150k	1.0	0.9	0.8	0.6	0.6	0.5	0.5	0.4	0.4	0.4	
	160k	0.9	0.8	0.7	0.6	0.5	0.5	0.4	0.4	0.4	0.4	
	170k	0.8	0.7	0.6	0.6	0.5	0.4	0.4	0.4	0.4	0.3	
	180k	0.8	0.7	0.6	0.5	0.5	0.4	0.4	0.4	0.3	0.3	
	190k	0.7	0.6	0.6	0.5	0.5	0.4	0.4	0.3	0.3	0.3	
	200k	0.7	0.6	0.5	0.5	0.4	0.4	0.4	0.3	0.3	0.4	

Table S14. Frame rate results of visualizing the 481 datasets using the D3-WebGL library.

Frame rate (fps)		Edge-to-node ratio										
		1	2	3	4	5	6	7	8	9	10	CG
Node scale	100	47.4	47.3	46.5	46.6	46.6	46.5	45.5	47.0	46.7	47.6	48.3
	200	45.0	46.7	45.9	46.5	45.8	44.8	45.4	45.8	47.3	47.1	48.4
	300	45.1	45.3	45.3	46.6	45.1	45.7	45.0	46.3	47.2	47.6	45.2
	400	46.5	45.2	45.2	45.9	45.0	45.1	45.1	45.6	48.2	46.6	44.8
	500	45.5	44.8	45.1	45.2	47.3	45.0	46.3	45.7	46.4	46.0	43.4
	600	45.8	45.0	45.1	44.3	46.4	45.8	46.2	46.0	45.4	46.2	42.1
	700	45.3	45.2	44.9	44.7	45.8	45.3	45.4	46.3	45.7	45.9	31.5
	800	45.4	45.2	44.9	45.6	45.7	46.5	46.3	46.4	47.2	45.5	27.3
	900	45.5	45.2	45.1	46.0	45.4	46.2	45.4	45.4	45.8	46.0	24.0
	1k	45.1	47.1	44.8	46.5	46.7	46.2	45.0	44.6	47.3	47.3	20.6
	2k	45.0	44.8	45.3	45.8	45.6	46.9	45.3	45.0	46.9	47.4	
	3k	44.9	45.1	43.8	47.1	44.6	43.8	43.9	44.4	47.6	47.2	

Frame rate (fps)		Edge-to-node ratio										
		**1**	**2**	**3**	**4**	**5**	**6**	**7**	**8**	**9**	**10**	**CG**
**Node scale**	**4k**	44.0	41.4	39.7	43.2	44.6	39.4	38.0	37.3	45.9	45.3	
	**5k**	42.4	32.6	32.8	36.7	43.9	33.4	33.6	33.7	37.4	39.9	
	**6k**	36.2	30.6	28.3	32.8	34.3	30.6	29.8	27.4	32.8	28.6	
	**7k**	30.6	27.1	24.2	28.2	28.1	26.7	26.1	24.6	29.1	26.1	
	**8k**	26.5	23.7	22.1	24.2	23.4	22.4	25.0	24.6	26.6	24.9	
	**9k**	25.1	23.6	23.5	23.0	22.4	20.6	20.1	18.3	20.0	24.6	
	**10k**	23.3	23.4	23.4	21.9	22.3	20.7	18.1	17.0	19.3	20.2	
	**15k**	14.3	12.9	13.3	14.8	12.1	14.0	13.7	11.6	12.8	11.7	
	**20k**	10.4	11.1	11.0	10.5	10.9	10.7	10.5	9.3	9.7	8.9	
	**25k**	7.2	9.0	7.8	7.6	7.9	8.2	8.0	7.5	7.6	6.3	
	**30k**	6.7	7.1	6.9	6.8	5.8	6.4	6.4	5.1	6.4	6.3	
	**35k**	5.6	6.0	5.7	5.7	4.5	5.2	5.2	4.1	5.3	5.0	
	**40k**	5.1	4.9	4.8	4.6	3.7	4.4	4.5	3.2	4.3	4.3	
	**45k**	4.3	4.4	4.0	3.9	4.0	3.9	3.8	3.8	3.5	3.6	
	**50k**	3.9	3.8	3.5	3.6	3.7	3.4	3.3	3.2	3.2	3.2	
	**55k**	3.4	3.4	3.0	2.9	3.2	3.0	2.7	2.9	2.8	2.8	
	**60k**	3.2	3.0	2.6	2.9	2.7	2.7	2.4	2.7	2.5	2.4	
	**65k**	2.7	2.7	2.4	2.4	2.4	2.3	2.4	2.4	2.3	2.2	
	**70k**	2.6	2.5	2.1	2.4	2.1	2.2	2.2	2.2	2.1	1.9	
	**75k**	2.2	2.3	2.0	2.2	2.1	2.0	2.0	1.8	1.9	1.7	
	**80k**	2.2	2.1	1.8	2.0	1.8	1.8	1.9	1.9	1.9	1.7	
	**85k**	1.9	2.0	1.6	1.8	1.7	1.6	1.6	1.8	1.6	1.5	
	**90k**	1.8	1.8	1.6	1.7	1.5	1.6	1.6	1.6	1.5	1.6	
	**95k**	1.6	1.7	1.4	1.6	1.6	1.6	1.4	1.5	1.4	1.3	
	**100k**	1.6	1.6	1.5	1.5	1.5	1.5	1.3	1.3	1.3	1.3	
	**110k**	1.4	1.4	1.3	1.2	1.3	1.2	1.1	1.3	1.1	0.9	
	**120k**	1.2	1.2	1.1	1.1	1.1	1.0	1.1	1.0	1.0	1.0	
	**130k**	1.1	1.1	1.0	0.9	1.0	0.9	0.9	0.9	0.9	0.8	
	**140k**	1.0	0.9	0.9	0.8	0.9	0.9	0.9	0.9	0.8	0.8	
	**150k**	0.9	0.8	0.9	0.8	0.8	0.8	0.8	0.8	0.7	0.7	
	**160k**	0.8	0.8	0.8	0.8	0.7	0.7	0.7	0.7	0.7	0.7	
	**170k**	0.7	0.8	0.7	0.7	0.6	0.7	0.5	0.6	0.6	0.6	
	**180k**	0.7	0.6	0.7	0.7	0.6	0.6	0.6	0.6	0.6	0.6	
	**190k**	0.7	0.6	0.6	0.6	0.5	0.6	0.6	0.6	0.5	0.6	
	**200k**	0.6	0.6	0.6	0.6	0.5	0.5	0.5	0.5	0.5	0.5	

Table S15. Frame rate results of visualizing the 481 graph datasets using the ECharts-Canvas library.

Frame rate (fps)		Edge-to-node ratio										
		**1**	**2**	**3**	**4**	**5**	**6**	**7**	**8**	**9**	**10**	**CG**
**Node scale**	**100**	59.2	58.6	59.9	59.4	59.4	59.6	60.0	57.7	58.4	58.8	57.8
	**200**	56.7	59.0	58.7	59.1	58.6	59.4	58.7	58.4	58.4	58.8	55.2
	**300**	59.1	58.7	58.8	59.6	56.3	57.4	57.7	58.6	58.4	56.1	27.8
	**400**	59.3	58.9	58.5	57.9	57.9	57.2	56.5	57.4	56.0	57.5	15.4
	**500**	58.5	58.0	58.5	57.4	57.4	58.1	57.4	56.5	56.0	57.0	10.7
	**600**	58.3	58.4	58.3	57.7	57.4	57.6	57.4	56.5	56.3	56.6	7.5
	**700**	59.0	58.5	58.1	57.8	56.9	57.1	56.2	55.7	54.9	55.4	5.3
	**800**	58.4	58.2	57.5	57.6	56.9	56.7	56.4	55.4	55.4	55.9	3.7
	**900**	58.3	58.2	57.7	57.1	56.3	56.5	56.5	56.2	55.1	55.5	3.1
	**1k**	58.6	58.2	56.9	57.4	56.8	56.7	56.1	55.1	54.6	55.5	2.5
	**2k**	57.3	55.6	48.8	45.0	42.1	38.1	33.8	33.2	33.0	32.9	null
	**3k**	31.9	27.7	24.9	22.4	22.1	21.1	19.3	18.5	17.9	17.8	
	**4k**	19.4	15.9	14.7	14.3	13.4	12.2	12.3	11.3	12.3	12.6	

Frame rate (fps)		Edge-to-node ratio										
		1	2	3	4	5	6	7	8	9	10	CG
Node scale	**5k**	12.1	11.0	10.1	9.8	9.8	9.1	8.8	8.7	8.8	8.8	
	**6k**	8.7	7.6	7.1	7.0	6.8	6.5	6.6	6.7	6.5	6.6	
	**7k**	6.5	5.6	5.5	5.4	5.2	5.1	5.0	4.6	5.1	5.1	
	**8k**	5.0	4.5	4.2	4.1	4.3	4.2	3.9	3.8	3.9	4.0	
	**9k**	4.1	3.3	3.5	3.5	3.6	3.3	3.1	3.3	3.2	3.3	
	**10k**	3.3	2.6	2.9	3.0	2.9	2.7	2.8	2.6	2.5	2.6	
	**15k**	1.3	1.3	1.2	1.2	1.4	1.3	1.3	1.2	1.2	1.2	
	**20k**	0.8	0.8	0.8	0.7	0.8	0.7	0.7	0.8	0.7	0.7	
	**25k**	0.6	0.5	0.5	0.5	0.5	0.5	0.5	0.5	0.5	0.5	
	**30k**	null	null	null	null	null	null	null	null	null	null	
	**35k**	null	null	null	null	null	null	null	null	null	null	
	**40k**	null	null	null	null	null	null	null	null	null	null	
	**45k**	null	null	null	null	null	null	null	null	null	null	
	**50k**	null	null	null	null	null	null	null	null	null	null	
	**55k**	null	null	null	null	null	null	null	null	null	null	
	**60k**	null	null	null	null	null	null	null	null	null	null	
	**65k**	null	null	null	null	null	null	null	null	null	null	
	**70k**	null	null	null	null	null	null	null	null	null	null	
	**75k**	null	null	null	null	null	null	null	null	null	null	
	**80k**	null	null	null	null	null	null	null	null	null	null	
	**85k**	null	null	null	null	null	null	null	null	null	null	
	**90k**	null	null	null	null	null	null	null	null	null	null	
	**95k**	null	null	null	null	null	null	null	null	null	null	
	**100k**	null	null	null	null	null	null	null	null	null	null	
	**110k**	null	null	null	null	null	null	null	null	null	null	
	**120k**	null	null	null	null	null	null	null	null	null	null	
	**130k**	null	null	null	null	null	null	null	null	null	null	
	**140k**	null	null	null	null	null	null	null	null	null	null	
	**150k**	null	null	null	null	null	null	null	null	null	null	
	**160k**	null	null	null	null	null	null	null	null	null	null	
	**170k**	null	null	null	null	null	null	null	null	null	null	
	**180k**	null	null	null	null	null	null	null	null	null	null	
	**190k**	null	null	null	null	null	null	null	null	null	null	
	**200k**	null	null	null	null	null	null	null	null	null	null	

Table S16. Frame rate results of visualizing the 481 datasets using the ECharts-SVG library.

Frame rate (fps)		Edge-to-node ratio										
		1	2	3	4	5	6	7	8	9	10	CG
Node scale	**100**	60.9	56.5	54.7	53.9	48.2	51.5	49.2	53.7	54.4	59.5	41.4
	**200**	60.0	59.0	53.7	51.0	48.4	53.9	52.6	53.9	54.3	53.0	11.8
	**300**	59.3	58.7	50.9	50.7	49.1	53.2	50.4	52.7	54.1	57.1	5.0
	**400**	59.3	58.9	52.1	51.6	51.0	52.9	52.3	50.8	50.0	50.9	2.8
	**500**	56.4	51.6	52.6	53.8	53.6	52.4	50.8	51.6	47.9	39.7	1.7
	**600**	58.8	52.8	51.3	53.6	52.3	51.2	45.4	39.6	37.0	34.6	1.1
	**700**	58.5	52.5	53.1	51.9	49.4	43.2	36.8	36.2	34.7	32.2	0.7
	**800**	56.6	54.3	51.8	50.0	47.7	36.3	33.9	31.3	28.5	26.2	0.6
	**900**	56.9	54.0	52.8	46.8	39.7	34.6	32.6	28.7	27.5	24.7	null
	**1k**	58.7	54.2	52.7	44.5	37.2	33.6	28.4	27.4	24.2	22.5	null
	**2k**	37.6	28.9	23.8	20.5	17.4	15.4	13.1	12.2	11.2	10.5	null
	**3k**	21.2	16.5	11.4	11.5	10.5	8.6	8.3	7.5	6.5	6.6	
	**4k**	13.0	10.6	7.7	7.6	7.3	6.3	5.6	5.4	4.9	4.7	
	**5k**	9.0	7.7	5.3	5.5	5.3	4.6	4.1	4.0	3.7	3.6	

Frame rate (fps)		Edge-to-node ratio										
		1	2	3	4	5	6	7	8	9	10	CG
Node scale	6k	6.7	5.8	3.9	4.3	4.1	3.6	3.4	3.0	3.0	2.8	
	7k	5.3	4.4	3.2	3.4	3.2	3.0	2.7	2.5	2.4	2.4	
	8k	4.2	3.3	2.5	2.7	2.6	2.4	2.1	1.9	1.8	1.9	
	9k	3.3	2.8	2.1	2.3	2.2	2.0	1.8	1.7	1.5	1.6	
	10k	2.5	2.3	2.1	2.1	1.9	1.7	1.6	1.5	1.3	1.3	
	15k	1.1	1.1	1.0	1.0	1.0	0.9	0.8	0.7	0.7	0.7	
	20k	0.7	0.7	0.6	0.7	0.6	0.6	0.5	0.5	0.6	0.6	
	25k	0.5	0.5	0.5	null	null	null	null	null	null	null	
	30k	null	null	null	null	null	null	null	null	null	null	
	35k	null	null	null	null	null	null	null	null	null	null	
	40k	null	null	null	null	null	null	null	null	null	null	
	45k	null	null	null	null	null	null	null	null	null	null	
	50k	null	null	null	null	null	null	null	null	null	null	
	55k	null	null	null	null	null	null	null	null	null	null	
	60k	null	null	null	null	null	null	null	null	null	null	
	65k	null	null	null	null	null	null	null	null	null	null	
	70k	null	null	null	null	null	null	null	null	null	null	
	75k	null	null	null	null	null	null	null	null	null	null	
	80k	null	null	null	null	null	null	null	null	null	null	
	85k	null	null	null	null	null	null	null	null	null	null	
	90k	null	null	null	null	null	null	null	null	null	null	
	95k	null	null	null	null	null	null	null	null	null	null	
	100k	null	null	null	null	null	null	null	null	null	null	
	110k	null	null	null	null	null	null	null	null	null	null	
	120k	null	null	null	null	null	null	null	null	null	null	
	130k	null	null	null	null	null	null	null	null	null	null	
	140k	null	null	null	null	null	null	null	null	null	null	
	150k	null	null	null	null	null	null	null	null	null	null	
	160k	null	null	null	null	null	null	null	null	null	null	
	170k	null	null	null	null	null	null	null	null	null	null	
	180k	null	null	null	null	null	null	null	null	null	null	
	190k	null	null	null	null	null	null	null	null	null	null	
	200k	null	null	null	null	null	null	null	null	null	null	

Table S17. Frame rate results of visualizing the 481 datasets using the G6-Canvas library.

Frame rate (fps)		Edge-to-node ratio										
		1	2	3	4	5	6	7	8	9	10	CG
Node scale	100	60.0	59.9	59.6	59.5	58.9	58.6	58.4	56.0	58.3	55.6	14.4
	200	57.6	59.1	58.0	58.2	57.6	51.0	44.7	41.1	35.0	31.3	3.2
	300	60.1	58.0	56.9	49.8	41.6	34.3	28.7	26.7	23.8	21.6	1.7
	400	59.3	57.9	40.8	35.1	29.5	25.2	20.8	20.5	17.7	16.2	0.9
	500	59.1	47.8	36.7	27.3	23.5	19.4	16.0	15.9	14.2	13.0	0.6
	600	57.5	40.3	31.1	24.3	19.9	17.0	14.8	13.1	11.4	10.6	0.4
	700	53.3	34.4	26.2	20.8	17.0	14.5	12.8	11.2	9.8	8.8	0.3
	800	50.0	30.3	23.3	18.4	14.9	12.4	10.6	9.5	8.5	7.8	null
	900	43.3	27.0	20.5	15.8	13.6	11.2	9.8	8.7	7.5	6.9	null
	1k	39.6	25.4	18.0	14.7	12.2	10.4	8.6	7.8	7.1	6.8	null
	2k	18.6	12.4	9.2	7.1	6.0	5.0	4.3	4.1	3.6	3.4	null
	3k	12.8	7.9	5.8	4.8	3.9	3.4	3.0	2.6	2.3	2.2	
	4k	9.7	6.0	4.5	3.6	3.0	2.6	2.1	1.9	1.9	1.8	
	5k	7.4	4.7	3.5	2.9	2.3	1.9	1.5	1.5	1.5	1.4	
	6k	6.2	3.8	3.0	2.4	1.9	1.7	1.3	1.3	1.3	1.2	

Frame rate (fps)		Edge-to-node ratio										
		1	2	3	4	5	6	7	8	9	10	CG
Node scale	**7k**	5.8	3.4	2.6	2.0	1.6	1.4	1.2	1.1	1.1	1.0	
	**8k**	4.9	2.9	2.2	1.7	1.5	1.2	1.1	0.9	0.9	0.9	
	**9k**	4.4	2.6	2.0	1.5	1.3	1.1	0.9	0.8	0.7	0.8	
	**10k**	4.1	2.4	1.8	1.4	1.2	1.0	0.8	0.7	0.7	0.6	
	**15k**	2.6	1.6	1.2	0.9	0.8	0.7	0.6	0.5	0.4	0.4	
	**20k**	2.2	1.2	0.8	0.7	0.6	0.5	0.4	0.3	0.3	0.3	
	**25k**	1.7	0.9	0.7	0.5	0.4	0.4	0.3	0.3	0.2	0.2	
	**30k**	1.5	0.8	0.6	0.4	0.4	0.3	0.3	0.2	null	null	
	**35k**	1.2	0.6	0.5	0.3	0.3	0.3	null	null	null	null	
	**40k**	1.1	0.6	0.4	0.3	0.3	0.2	null	null	null	null	
	**45k**	0.9	0.5	0.4	0.3	0.2	null	null	null	null	null	
	**50k**	0.9	0.5	0.3	0.2	null	null	null	null	null	null	
	**55k**	0.8	0.4	0.3	0.2	null	null	null	null	null	null	
	**60k**	0.7	0.4	0.3	null	null	null	null	null	null	null	
	**65k**	0.6	0.4	0.3	null	null	null	null	null	null	null	
	**70k**	0.6	0.3	0.2	null	null	null	null	null	null	null	
	**75k**	0.5	0.3	null	null	null	null	null	null	null	null	
	**80k**	0.5	0.3	null	null	null	null	null	null	null	null	
	**85k**	0.5	0.3	null	null	null	null	null	null	null	null	
	**90k**	0.4	0.3	null	null	null	null	null	null	null	null	
	**95k**	0.4	0.2	null	null	null	null	null	null	null	null	
	**100k**	0.4	null	null	null	null	null	null	null	null	null	
	**110k**	0.4	null	null	null	null	null	null	null	null	null	
	**120k**	0.3	null	null	null	null	null	null	null	null	null	
	**130k**	0.3	null	null	null	null	null	null	null	null	null	
	**140k**	0.3	null	null	null	null	null	null	null	null	null	
	**150k**	0.2	null	null	null	null	null	null	null	null	null	
	**160k**	null	null	null	null	null	null	null	null	null	null	
	**170k**	null	null	null	null	null	null	null	null	null	null	
	**180k**	null	null	null	null	null	null	null	null	null	null	
	**190k**	null	null	null	null	null	null	null	null	null	null	
	**200k**	null	null	null	null	null	null	null	null	null	null	

Table S18. Frame rate results of visualizing the 481 datasets using the G6-SVG library.

Frame rate (fps)		Edge-to-node ratio										
		1	2	3	4	5	6	7	8	9	10	CG
Node scale	**100**	60.1	59.8	53.6	53.0	42.3	49.0	44.2	37.8	35.6	35.8	7.8
	**200**	59.3	49.0	41.5	32.9	31.4	25.5	22.4	21.2	18.4	16.5	1.9
	**300**	58.6	37.9	29.7	23.8	21.7	17.2	15.0	14.3	12.5	11.9	0.8
	**400**	49.6	29.3	20.7	19.0	16.2	13.5	11.9	10.6	9.3	8.9	0.5
	**500**	39.1	23.9	19.9	15.0	12.8	10.7	9.4	8.5	7.6	7.1	0.3
	**600**	33.0	21.4	16.3	12.9	10.7	8.9	7.9	7.1	6.2	5.8	null
	**700**	27.3	18.9	13.7	10.8	8.9	7.7	6.8	5.9	5.3	5.0	null
	**800**	24.3	16.0	11.8	9.7	8.1	6.7	5.9	5.2	4.6	4.3	null
	**900**	23.0	14.4	10.7	8.5	7.3	6.0	5.3	4.6	4.1	3.8	null
	**1k**	21.0	13.5	9.8	7.8	6.4	5.5	4.7	4.2	3.8	3.5	null
	**2k**	10.0	6.5	4.7	3.7	3.1	2.7	2.2	2.1	1.8	1.7	null
	**3k**	6.8	4.2	3.1	2.4	2.0	1.7	1.5	1.4	1.2	1.1	
	**4k**	4.9	3.1	2.3	1.8	1.6	1.3	1.1	1.0	0.9	0.9	
	**5k**	4.0	2.5	1.9	1.5	1.2	1.0	0.9	0.8	0.7	0.7	
	**6k**	3.3	2.1	1.5	1.2	1.0	0.8	0.8	0.7	0.6	0.6	
	**7k**	2.7	1.8	1.3	1.0	0.9	0.7	0.6	0.6	0.5	0.5	

Frame rate (fps)		Edge-to-node ratio										
		**1**	**2**	**3**	**4**	**5**	**6**	**7**	**8**	**9**	**10**	**CG**
**Node scale**	**8k**	2.4	1.5	1.1	0.9	0.8	0.6	0.6	0.5	0.5	0.4	
	**9k**	2.2	1.4	1.0	0.8	0.7	0.6	0.5	0.4	0.4	0.4	
	**10k**	2.0	1.3	0.9	0.7	0.6	0.5	0.4	0.4	0.4	0.3	
	**15k**	1.3	0.8	0.6	0.5	0.4	0.3	0.3	0.3	null	null	
	**20k**	0.9	0.6	0.5	0.4	0.3	0.3	null	null	null	null	
	**25k**	0.8	0.5	0.4	0.3	null	null	null	null	null	null	
	**30k**	0.6	0.4	0.3	null	null	null	null	null	null	null	
	**35k**	0.5	0.3	null	null	null	null	null	null	null	null	
	**40k**	0.5	0.3	null	null	null	null	null	null	null	null	
	**45k**	0.4	null	null	null	null	null	null	null	null	null	
	**50k**	0.4	null	null	null	null	null	null	null	null	null	
	**55k**	0.3	null	null	null	null	null	null	null	null	null	
	**60k**	0.3	null	null	null	null	null	null	null	null	null	
	**65k**	0.3	null	null	null	null	null	null	null	null	null	
	**70k**	null	null	null	null	null	null	null	null	null	null	
	**75k**	null	null	null	null	null	null	null	null	null	null	
	**80k**	null	null	null	null	null	null	null	null	null	null	
	**85k**	null	null	null	null	null	null	null	null	null	null	
	**90k**	null	null	null	null	null	null	null	null	null	null	
	**95k**	null	null	null	null	null	null	null	null	null	null	
	**100k**	null	null	null	null	null	null	null	null	null	null	
	**110k**	null	null	null	null	null	null	null	null	null	null	
	**120k**	null	null	null	null	null	null	null	null	null	null	
	**130k**	null	null	null	null	null	null	null	null	null	null	
	**140k**	null	null	null	null	null	null	null	null	null	null	
	**150k**	null	null	null	null	null	null	null	null	null	null	
	**160k**	null	null	null	null	null	null	null	null	null	null	
	**170k**	null	null	null	null	null	null	null	null	null	null	
	**180k**	null	null	null	null	null	null	null	null	null	null	
	**190k**	null	null	null	null	null	null	null	null	null	null	
	**200k**	null	null	null	null	null	null	null	null	null	null	
